# Extraction Methods Shape the Phenolic Composition and Bioactivities of Defatted Moroccan *Pistacia lentiscus* L. Resin

**DOI:** 10.3390/antiox14101207

**Published:** 2025-10-05

**Authors:** Abdessamad Beraich, Daniela Batovska, Krastena Nikolova, Burak Dikici, Göksen Gören, Yousra Belbachir, Mohamed Taibi, Amine Elbouzidi, Irena Mincheva, Natalina Panova, Abdesselam Tahani, Abdeslam Asehraou, Abdelmonaem Talhaoui

**Affiliations:** 1 Environment and Applied Chemistry (LCAE), Team: Physical Chemistry of the Natural Resources and Processes, Department of Chemistry, Faculty of Sciences, Mohamed First University, Oujda 60000, Morocco; abdessamad.beraich@ump.ac.ma (A.B.); yousra.belbachir@ump.ac.ma (Y.B.); a1.tahani@ump.ac.ma (A.T.); a.talhaoui@ump.ac.ma (A.T.); 2 Department of Mechanical Engineering, Faculty of Engineering, Ataturk University, 25240 Erzurum, Turkey; burakdikici@gmail.com; 3 Institute of Chemical Engineering, Bulgarian Academy of Sciences, Acad. G. Bonchev Str., Bl. 103, 1113 Sofia, Bulgaria; irenamincheva@gmail.com; 4 Department of Physics and Biophysics, Faculty of Pharmacy, Medical University of Varna, 9000 Varna, Bulgaria; panova@mu-varna.bg; 5 Department of Pathology Laboratory Techniques, Vocational School of Health Services, İstinye University, 34156 Istanbul, Turkey; goksen.goren16@ogr.atauni.edu.tr; 6 Laboratory of Agricultural Production Improvement, Biotechnology, and Environment (LAPABE), Faculty of Sciences, Mohammed First University, Oujda 60000, Morocco; mohamedtaibi9@hotmail.fr (M.T.); amine.elbouzidi@ump.ac.ma (A.E.); 7 Laboratory of Bioresources Biotechnology, Ethnopharmacology, and Health, Team: Microbiology, Faculty of Sciences, Mohammed First University, Oujda 60000, Morocco; a.asehraou@ump.ac.ma

**Keywords:** *Anacardiaceae*, antibacterial activity, antifungal activity, antioxidant activity, cytotoxicity, mastic gum, phenolics, solvent polarity, ultrasound-assisted extraction

## Abstract

Mastic gum from *Pistacia lentiscus* L. has long been valued in Mediterranean medicine and food preservation, yet its bioactive potential remains underexplored in specific geographic contexts. In Morocco, the resin—locally known as Meska Horra—is abundant but insufficiently characterized. This study compared three extraction methods—cold maceration (CM), Soxhlet extraction (SE), and ultrasound-assisted extraction (UAE)—using sequential acetone and 70% ethanol to recover complementary phenolic compounds from defatted resin. Targeted UHPLC–ESI–MS/MS profiling identified and quantified 30 phenolics, mainly flavonoids and phenolic acids, providing the first systematic dataset for Moroccan mastic gum. UAE–EtOH extract displayed the strongest antioxidant activity (DPPH IC_50_ = 0.029 mg/mL; ABTS•^+^ IC_50_ = 0.026 mg/mL). SE–acetone and SE–EtOH extracts showed potent antifungal activity, particularly against *Geotrichum candidum*, *Rhodotorula glutinis*, and *Aspergillus niger* (MBC = 1.7%). The SE–acetone extract exhibited cytotoxicity toward MIA PaCa-2 pancreatic cancer cells (IC_50_ = 19 µg/mL). These findings demonstrate that extraction method and solvent choice strongly influence phenolic recovery and associated bioactivities, supporting the valorization of Moroccan mastic gum as a promising source for nutraceutical and pharmaceutical applications.

## 1. Introduction

Mastic gum, the aromatic resin exuded by *Pistacia lentiscus* L. (*Anacardiaceae*), has been used for centuries across the Mediterranean region in traditional medicine, culinary, and sensorial applications [1,2]. Its functional significance is primarily attributed to terpenoids and volatiles, which are known to possess antioxidant, antimicrobial, anti-inflammatory, and cytoprotective properties [3,4,5]. Among regional sources, the mastic gum produced on the Greek island of Chios is the most thoroughly characterized and holds Protected Designation of Origin (PDO) status within the European Union [2,6]. Due to limited supply and rising global demand, recent research has focused on alternative production areas with similar ecological conditions, such as the Çeşme Peninsula in Türkiye [7,8,9].

In Morocco, *P. lentiscus* L. grows wild and abundantly, particularly in the northern and eastern regions of the country [10]. The resin, locally known as *Meska Horra*, is traditionally harvested for artisanal use but remains largely underutilized due to the absence of industrial development and limited research [11]. While most studies have focused on the leaves [12,13,14,15], recent data indicate that the resin’s essential oil meets European Pharmacopoeia standards and displays notable antioxidant and antimicrobial activity [11], highlighting its potential as a promising natural source of bioactive compounds.

Still, these findings represent only an initial step toward understanding the resin’s full potential [11]. To date, no systematic study has explored the recovery of bioactive compounds from Moroccan mastic gum beyond its essential oil, nor evaluated how different extraction methods influence its phytochemical composition and associated bioactivities. The absence of optimized protocols for selectively enhancing specific biological activities further limits its broader application and industrial development [16,17,18].

Accumulated research on *P. lentiscus* L. leaves and mastic gum from other geographic regions has demonstrated that combining phytochemical profiling with biological assays is an effective strategy for identifying functionally relevant compounds [8,12,13,15,19,20,21]. These studies also emphasize the importance of selecting appropriate extraction methods to preserve and concentrate bioactive constituents [22,23]. Phenolic profiling of mastic gum has been addressed in only a single published investigation. In that work, maceration with acetone, methanol, and ethanol revealed salicylic, rosmarinic, and caffeic acids as the predominant phenolic constituents, while parallel targeted analyses identified additional flavonoids and phenolic acids, including rutin, luteolin-7-glucoside, apigenin-7-glucoside, naringenin, and ellagic acid [8]. More recently, studies on *P. atlantica* subsp. kurdica mastic gum linked antioxidant and cytotoxic activities to total phenolic content, although individual compounds were not resolved [20]. Despite these advances, the phenolic fraction of the resin has received far less attention than its well-documented terpenoid composition [19,20,21]. Comprehensive evaluations integrating phenolic diversity with biological activity are therefore required to clarify their contribution to resin bioactivity and to support the targeted valorization of Moroccan resin for nutraceutical, cosmetic, and therapeutic applications.

Accordingly, this study aims to identify effective extraction strategies for maximizing the bioactive potential of Moroccan *P. lentiscus* L. resin. To this end, we applied a sequential extraction approach (acetone → 70% ethanol) on defatted resin, enabling polarity-guided recovery of complementary metabolite pools. Particular focus is placed on phenolic compounds due to their well-documented contributions to the antioxidant, antimicrobial, and cytotoxic profiles in medicinal plants [24,25].

## 2. Materials and Methods

### 2.1. Chemicals and Materials

All reagents and solvents used in this study were of analytical grade and were purchased from Sigma-Aldrich (St. Louis, MO, USA), unless otherwise specified. Analytical standards used for compound quantification had purities of ≥95%.

The bacterial strains *Staphylococcus aureus* (ATCC 25923), *Micrococcus luteus* (ATCC 4698), *Escherichia coli* (ATCC 25922), and *Pseudomonas. aeruginosa* (ATCC 27853), as well as the fungal strains *Geotrichum candidum* (ATCC 34614), *Aspergillus niger* (ATCC 16404), *Candida glabrata* (ATCC 90030), *Candida albicans* (ATCC 10231), and *Rhodotorula glutinis* (ATCC 204091) were obtained from the American Type Culture Collection (ATCC, Manassas, VA, USA).

The WST-8 Cell Proliferation Assay Kit was purchased from Dojindo Molecular Technologies (Kumamoto, Japan). The human pancreatic cancer cell line MIA PaCa-2 (ATCC^®^ CRL-1420^TM^) was obtained from the American Type Culture Collection (ATCC, Manassas, VA, USA).

Cell culture reagents, including Dulbecco’s Modified Eagle Medium (DMEM), fetal bovine serum (FBS), and penicillin–streptomycin solution, were obtained from Gibco, Thermo Fisher Scientific (Waltham, MA, USA).

### 2.2. Plant Material

The resin of *P. lentiscus* L. was collected from wild-growing plants in the eastern region of Morocco (34°31′04″ N, 1°50′35″ W) by making deliberate incisions into the main stem, allowing the resin to naturally exude and harden. A voucher specimen was authenticated and deposited in the herbarium of Université Mohamed Premier in Oujda, Morocco, under accession number UMPOM782.

After collection, the resin was carefully washed with distilled water to remove dust and adhering particles, then dried in a ventilated oven at 25–32 °C for seven days, a range chosen to ensure adequate dehydration while preserving thermolabile constituents such as phenolic compounds. The dried material was ground into a fine powder and stored under dry conditions. Immediately prior to extraction, the powdered resin underwent a defatting step designed to minimize non-polar interference and enhance the recovery of phenolic compounds. This was performed by maceration with *n*-hexane (1:10 *w*/*v*) for 24 h at room temperature with occasional stirring. The *n*-hexane phase was decanted, and the process was repeated twice. The defatted material was then air-dried to remove residual solvent.

The total amount of resin obtained was limited by the seasonal and manual nature of collection, providing only enough material for the present extractions and analyses.

### 2.3. Extraction of Resin

#### 2.3.1. Extraction Methods

To evaluate the effect of extraction method and solvent polarity on the chemical composition and biological activity of defatted *P. lentiscus* L. resin, acetone (100%) and ethanol (EtOH, 70% *v*/*v*) were applied sequentially in each extraction protocol.

The extraction methods were performed as follows:

- Ultrasound-Assisted Extraction (UAE): 20 g of powdered resin were extracted sequentially with 200 mL of each solvent in a WiseClean WUC-D22H ultrasonic bath (230 V, 50 Hz; frequency: 60 Hz; power: 500 W) at 40 °C for 15 min per solvent. Extraction time was selected according to commonly reported UAE protocols for phenolic compounds (typically 10–20 min) to ensure efficient recovery [26,27].
- Soxhlet Extraction (SE): 20 g of powdered resin were extracted sequentially with 200 mL of each solvent using a Soxhlet apparatus under reflux for 4 h per solvent cycle. A 4 h duration was selected as standard for phenolic recovery in Soxhlet extraction [24,28].
- Cold Maceration (CM): 20 g of powdered resin were mixed with 200 mL of solvent and stirred at ambient (room) temperature (100 rpm) in the dark for 24 h using a magnetic stirrer. A 24 h maceration period is commonly employed to ensure adequate recovery of phenolic compounds [29,30]. After filtration and solvent evaporation, the dried residue was subjected to the next solvent in the polarity sequence.

All resulting extracts were filtered, concentrated under reduced pressure using a rotary vacuum evaporator, and stored at 4 °C in the dark until further analysis.

#### 2.3.2. Extraction Yield

The extraction yield was calculated as the percentage of dry extract obtained relative to the initial dry mass of the resin, according to the following equation:

$$Yield\;\left(\%\right)=\left(\frac{Mass\;of\;extract\;(g)}{Mass\;of\;raw\;material\;\left(g\right)}\right)\;\times 100$$

Extractions were performed once per solvent–method combination due to limited resin availability; therefore, yield values are indicative only and were not statistically analyzed.

### 2.4. Phytochemical Analyses

#### 2.4.1. Quantification of Total Phenolic Content (TPC) and Total Flavonoid Content (TFC)

The TPC and TFC of the extracts were determined using standard colorimetric assays, following the method of Beraich et al. [19], with slight modifications. All measurements were performed in triplicate.

TPC was measured using the Folin–Ciocalteu method. Briefly, 100 µL of each extract (0.5 mg/mL in methanol) was mixed with 400 µL of 1:10 diluted Folin–Ciocalteu reagent and 100 µL of distilled water. After 5 min, 2.4 mL of 20% sodium carbonate solution was added. The reaction mixture was incubated in the dark at room temperature for 1 h, and absorbance was measured at 760 nm.

TFC was determined using the aluminum chloride colorimetric method. A reagent solution was prepared by dissolving 133 mg of aluminum chloride and 430 mg of sodium acetate in 100 mL of distilled water. Each extract (1 mL, 0.5 mg/mL in methanol) was mixed with 500 µL of the reagent solution and incubated at room temperature for 30 min. Absorbance was measured at 430 nm.

Calibration curves were constructed for gallic acid and quercetin using six standard concentrations ranging from 10 to 100 µg/mL. The results showed excellent linearity, with R^2^ values of 0.9992 for TPC and 0.9987 for TFC.

All absorbance measurements were recorded using a MULTISKAN GO microplate spectrophotometer (version 1.00.40, Thermo Fisher Scientific, Waltham, MA, USA). Results were expressed as milligrams of gallic acid equivalents per gram of extract (mg GAE/g) for TPC and milligrams of quercetin equivalents per gram of extract (mg QE/g) for TFC.

#### 2.4.2. Phenolic Compound Profiling

The identification and quantification of individual phenolic compounds in the resin extracts were performed using ultra-high-performance liquid chromatography coupled with electrospray ionization tandem mass spectrometry (UHPLC–ESI–MS/MS). Each extract was dissolved in methanol at a concentration of 10 mg/mL prior to analysis.

The UHPLC system (Agilent 1290 Infinity Series, Agilent Technologies, Santa Clara, CA, USA) was equipped with the following modules:

- Autosampler (G4226A).
- Sampler thermostat (G1330B).
- Quaternary pump with degasser (G4204A; 1200 bar capacity).
- Thermostated column compartment (G1316A).

Chromatographic separation was achieved using a reversed-phase analytical column (Zorbax SB-C18, 4.6 × 100 mm, 3.5 μm; Agilent Technologies, Santa Clara, CA, USA). The mobile phase consisted of solvent A (water with 0.1% formic acid) and solvent B (acetonitrile with 0.1% formic acid), using the following gradient program:

- 0 min: 5% B.
- 4 min: 20% B (linear increase from 0 to 4 min).
- 7 min: 90% B (linear increase from 4 to 7 min).
- 7–14 min: hold at 90% B.
- 14–15.1 min: decrease to 5% B.
- 15.1–20 min: re-equilibrate at 5% B.

The flow rate was 0.4 mL/min, and the column temperature was maintained at 30 °C.

Detection and quantification were performed on an Agilent 6460 triple quadrupole mass spectrometer equipped with an electrospray ionization (ESI) source operated in both positive and negative ion modes, depending on the analyte. The optimized source parameters were: capillary voltage, 3500 V (positive mode) and 3000 V (negative mode); gas temperature, 300 °C; drying gas flow, 10 L/min; nebulizer pressure, 45 psi; sheath gas temperature, 350 °C; sheath gas flow, 11 L/min. Multiple reaction monitoring (MRM) mode was used, in which compound-specific precursor and product ion transitions are monitored for each analyte. The fragmentor voltage was set at 135 V, and collision energies ranged from 10 to 35 eV, optimized for each transition. Transitions, collision energies, and ion modes for all analytes are provided in Supplementary Table S1. Data acquisition and processing were conducted using MassHunter Qualitative and Quantitative Analysis software (version B07; Agilent Technologies, Santa Clara, CA, USA).

Identification followed MSI Level 1 criteria using authentic standards (*n* = 30) analyzed under identical conditions. Retention times were matched within Δt_R_ < 0.05 min, and compound-specific MRM transitions (precursor and product ions) corresponded exactly to those of the standards. Quantification was performed using individual seven-point calibration curves for each standard (100–2000 µg/L; R^2^ > 0.99). Quality control (QC) samples were prepared at three concentration levels to verify accuracy: 250, 750, and 1500 µg/L for caffeic acid and *p*-hydroxybenzoic acid, and 100, 750, and 1500 µg/L for all other analytes. The slightly higher low QC level for caffeic acid and *p*-hydroxybenzoic acid was selected to improve measurement precision for these analytes at the lower end of their calibration ranges.

Total ion chromatograms (TICs) for all extracts are provided in Figure S1 to enable visual comparison of profiles across extraction methods and solvents.

### 2.5. Biological Activity Evaluation

#### 2.5.1. Antioxidant Activity

The antioxidant potential of the resin extracts was evaluated using two complementary in vitro assays: the 2,2-diphenyl-1-picrylhydrazyl (DPPH) radical scavenging assay and the 2,2′-azino-bis(3-ethylbenzothiazoline-6-sulfonic acid (ABTS•^+^) decolorization assay. Both methods assess the extracts’ ability to neutralize free radicals and thus reflect their antioxidant efficacy. All absorbance measurements were performed in triplicate using the microplate spectrophotometer described in Section 2.5.1.

The DPPH radical scavenging assay was conducted following a modified procedure by Beraich et al. [19]. Briefly, 2400 µL of 0.1 mM DPPH solution in EtOH was mixed with 600 µL of extract at various concentrations (0.004–0.2 mg/mL). The mixtures were incubated in the dark at room temperature for 60 min, and absorbance was measured at 517 nm.

The ABTS•^+^ assay was based on the method of Re et al. [20], with slight modifications. ABTS•^+^ radicals were generated by mixing 9.5 mL of 0.7 M ABTS with 0.245 mL of 0.1 M potassium persulfate and allowing the solution to stand in the dark at room temperature for 18 h. Prior to analysis, the solution was diluted with 0.1 M potassium phosphate buffer (pH 7.4) to achieve an absorbance of 0.70 ± 0.02 at 734 nm. Extracts were dissolved in EtOH across a concentration range of 0.004 to 0.2 mg/mL. In each reaction, 2400 µL of the ABTS•^+^ solution was mixed with 600 µL of extract, and absorbance was measured at 734 nm.

Radical scavenging activity was calculated using the following equation:

$$\%\;Inhibition=\left(\frac{Acontrol-Asample}{Acontrol}\right)\times 100,$$

where

- *Acontrol* is the absorbance of the radical solution without extract;
- *Asample* is the absorbance in the presence of extract.

IC_50_ values, defined as the concentration of extract required to scavenge 50% of radicals, were determined by plotting the inhibition percentage against concentration and fitting the data using non-linear regression (four-parameter logistic model). Ascorbic acid was used as a positive control for both assays.

#### 2.5.2. Antimicrobial Activity

The antimicrobial potential of the extracts was assessed against a panel of four bacterial and five fungal strains. The bacterial panel comprised two Gram-positive species (*S. aureus* and *M. luteus*) and two Gram-negative species (*E. coli* and *P. aeruginosa*). The fungal panel included three yeasts (*C. albicans*, *C. glabrata*, and *R. glutinis*) and two molds (*A. niger* and *G. candidum*).

Bacterial strains were maintained on Mueller–Hinton agar (MHA) and incubated at 37 °C, while fungal strains were cultured on potato dextrose agar (PDA) at 25 °C.

Antimicrobial screening was initially screened using the agar well diffusion method, following the procedure described by Balouiri et al. [31]. Microbial suspensions were standardized to 0.5 McFarland turbidity (≈1 × 10^8^ CFU/mL). For bacteria, 100 µL of the suspension was evenly spread on MHA plates (15 mL per plate) using sterile cotton swabs in three directions. Fungal inoculation was performed analogously on PDA plates. After a brief drying period (~5 min), wells of 6 mm diameter were aseptically punched into the agar using a sterile cork borer. Each well was filled with 50 µL of the test extract. Gentamicin (1 mg/mL) and cycloheximide (1 mg/mL) were used as positive controls for bacteria and fungi, respectively. All assays were conducted in triplicate.

After incubation (24 h at 37 °C for bacteria and 48 h at 25 °C for fungi), the diameters of the inhibition zones (including the well diameter) were measured in millimeters. Results were expressed as mean ± standard deviation from three independent experiments.

Minimum inhibitory concentrations (MICs) were determined using a 96-well microdilution method in media supplemented with 0.15% agar (MHA broth for bacteria, PDA broth for fungi). Extracts were serially diluted (16% to 0.0015% *v*/*v*), and each well was inoculated with microbial suspension. Following incubation under the same conditions, resazurin was added as a metabolic indicator; a color change from blue to pink indicated microbial viability [32]. Positive controls were included in all experiments.

Minimum bactericidal concentrations (MBCs) and minimum fungicidal concentrations (MFCs) were determined according to the method of Thosar et al. [33]. From wells showing no color change, 3 µL aliquots were plated on fresh MHA (for bacteria) or PDA (for fungi) and re-incubated (24 h for bacteria, 48 h for yeasts, and 72 h for molds). MBC and MFC values were defined as the lowest extract concentrations at which no visible growth was observed, indicating complete microbial inhibition.

#### 2.5.3. Cytotoxicity Evaluation via WST-8 Assay

The cytotoxic potential of the resin extracts was evaluated using the WST-8 assay, a colorimetric method that quantifies metabolically active cells based on the reduction in a tetrazolium salt to a water-soluble formazan dye. The anticancer assessment was performed exclusively on a human pancreatic carcinoma cell line (MIA PaCa-2). No non-tumorigenic control cell lines were included in this study.

MIA PaCa-2 cells were cultured in Dulbecco’s Modified Eagle Medium (DMEM) supplemented with 10% fetal bovine serum (FBS) and 1% penicillin–streptomycin under standard conditions (37 °C, 5% CO_2_, humidified atmosphere). Initially, cells were seeded into T25 flasks and incubated for 24 h, then subcultured into T75 flasks and grown to approximately 70% confluency. Cells were harvested, counted using a hemocytometer (Neubauer improved, BRAND GMBH, Wertheim, Germany), and seeded into 96-well plates at a density of 10,000–20,000 cells per well in 100 µL of complete medium. After a 24 h attachment period, cells were subjected to treatment.

Test extracts were first dissolved in 100 µL of dimethyl sulfoxide (DMSO) and diluted with 900 µL of DMEM to obtain a 1 mg/mL stock solution. Serial two-fold dilutions were performed to yield six final concentrations: 500, 250, 125, 62.5, 31.25, and 15.625 µg/mL. A 10% DMSO solution was used as a positive control, while untreated cells in complete DMEM served as the negative control. Cell viability in the negative control was defined as 100%, and the viability of treated groups was expressed as a percentage of this reference.

After 48 h of incubation with the extracts, WST-8 reagent was added to each well at 10% of the total volume. Plates were incubated in the dark, and absorbance was measured at 450 nm at 1, 2, and 3 h using a microplate reader (MULTISKAN GO, version 1.00.40, Thermo Fisher Scientific, Waltham, MA, USA). The degree of color development was directly proportional to the number of viable cells, enabling quantification of cytotoxicity in a dose-dependent manner. IC_50_ values were determined by non-linear regression analysis.

All assays, including absorbance measurements and IC_50_ calculations, were performed in triplicate to ensure data reliability and reproducibility.

### 2.6. Statistical Analysis

The experimental data were analyzed using appropriate statistical methods based on the nature of each dataset. Antioxidant, antimicrobial, and cytotoxicity assays were performed in triplicate, and results are expressed as mean ± standard deviation (SD). IC_50_ values were calculated by non-linear regression using a four-parameter logistic model implemented in Python (version 3.11.6) with the SciPy library.

Two-way analysis of variance (ANOVA) was applied to assess the effects of extraction method and solvent polarity on TPC, TFC, and biological activities. When significant main or interaction effects were detected (*p* < 0.05), Tukey’s post hoc test was applied for pairwise comparisons between groups. ANOVA and Tukey tests were performed in XLSTAT (version 2023.3.1; Addinsoft, Paris, France), with significance set at *p* < 0.05.

Multivariate analyses were performed to explore patterns with the phytochemical dataset. Principal component analysis (PCA) and agglomerative hierarchical clustering (AHC) were used to identify grouping among extracts. Clustered heatmaps and Spearman’s rank correlation matrices were generated visualized the distribution of phenolic compounds across extracts and their associations with biological activities. Fold-change analyses and bar plots illustrated differences in compound abundance among extraction conditions. Class-level comparisons were visualized using bar charts representing the mean z-score values of phenolic compound classes per extraction method. All visualizations were generated using XLSTAT (version 2023.3.1; Addinsoft, Paris, France), Python (version 3.11.6) and the Matplotlib (version 3.8.2) and Seaborn (version 0.13.2) libraries.

## 3. Results

### 3.1. Extraction Yields

**Figure 1:**
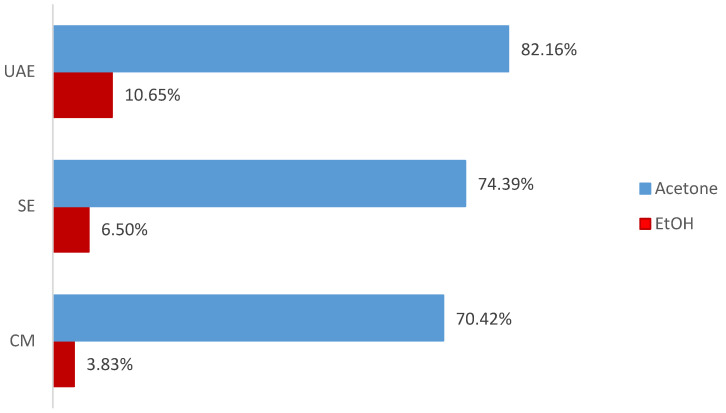
Extraction yields (%) of defatted *P. lentiscus* L. resin obtained by ultrasound-assisted extraction (UAE), Soxhlet extraction (SE), and cold maceration (CM) using acetone and 70% EtOH as sequential solvents. Values correspond to single extractions performed under each condition.

Sequential extraction of defatted *P. lentiscus* L. resin from Morocco was performed using acetone and 70% EtOH (*v*/*v*) in combination with CM, SE, and UAE. Acetone consistently produced higher yields than EtOH across all methods: 82% vs. 11% (UAE), 74% vs. 7% (SE), and 70% vs. 4% (CM) (Figure 1). UAE showed the highest efficiency among the tested techniques, emphasizing the advantage of ultrasound-assisted diffusion in resin matrix disruption.

### 3.2. Phytochemical Composition of the Resin Extracts

#### 3.2.1. TPC and TFC

**Figure 2:**
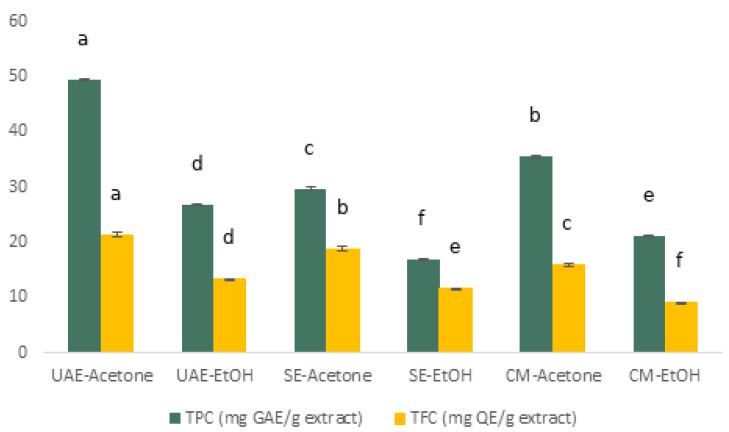
Total phenolic content (TPC) and total flavonoid content (TFC) of defatted *P. lentiscus* L. resin extracts obtained by UAE, SE, and CM using acetone and 70% EtOH as sequential solvents. Data are mean ± standard deviation (SD) from three independent extractions (*n* = 3). Different letters above bars indicate significant differences within each dataset (TPC or TFC) according to Tukey’s HSD test (α = 0.05). Letters are not comparable between TPC and TFC. Full ANOVA/Tukey in Tables S2–S5.

TPC and TFC of *P. lentiscus* L. resin extracts are summarized in Table S1 and illustrated in Figure 2. Acetone consistently yielded higher concentrations of both phenolics and flavonoids compared to 70% EtOH, with UAE extracts outperforming SE and CM.

Two-way ANOVA (method x solvent) showed significant main effects and a significant interaction for both TPC and TFC (all *p* ≤ 0.0017; Tables S2 and S3). Tukey’s HSD identified UAE–acetone as higher than each of the other five extracts for both endpoints (all adjusted *p* < 0.001; Tables S4 and S5).

#### 3.2.2. Phenolic Profiles

A total of 30 phenolic compounds—including phenolic acids and flavonoids—were identified and quantified by UHPLC–ESI–MS/MS (Figure S1, Table S6). All analytes were confirmed with authentic standards (MSI Level 1) and quantified using compound-specific calibration curves. Normalized concentrations were further subjected to z-score transformation for clustering.

**Figure 3:**
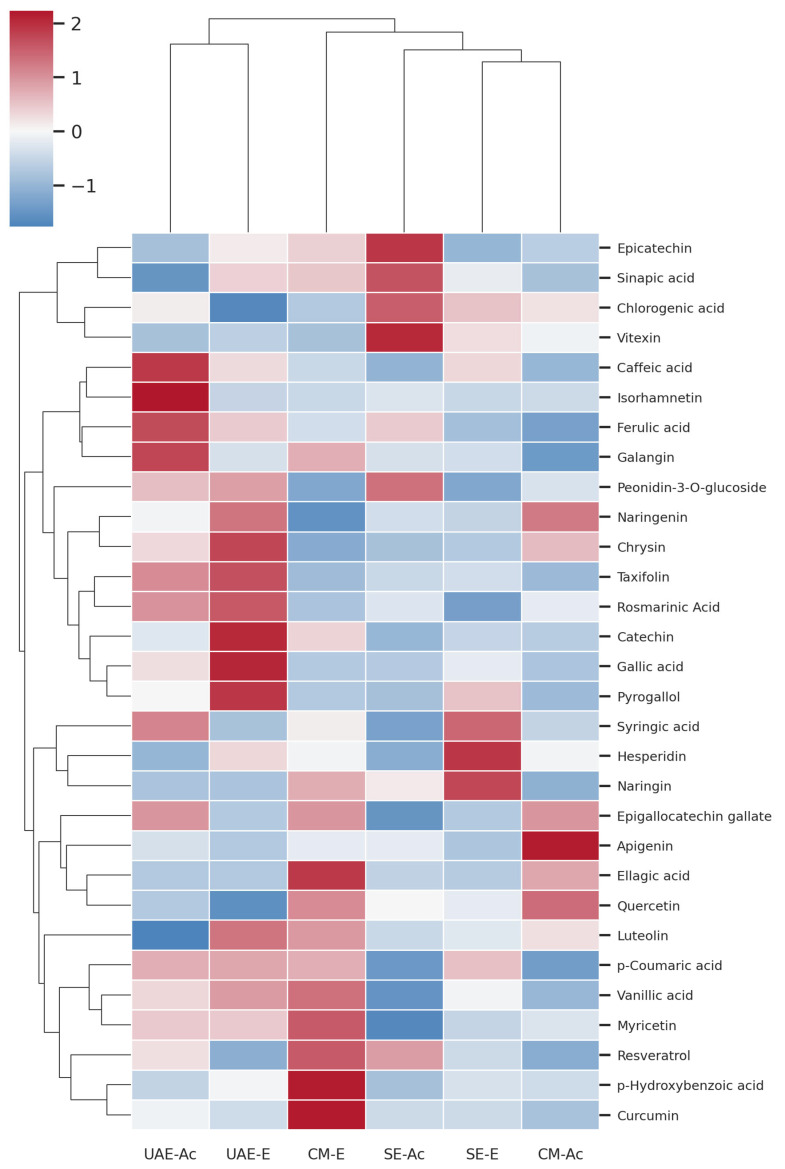
Hierarchical clustered heatmap of z-score normalized concentrations of phenolic compounds in *P. lentiscus* L. resin extracts—UAE-(Ac)etone, UAE-(E)thanol, CM-(E)thanol, SE-(Ac)etone, SE-(E)thanol, and CM-(Ac)etone.

A hierarchical clustering heatmap (Figure 3) revealed distinct phytochemical profiles across extracts, strongly influenced by both solvent and extraction method.

CM–EtOH exhibited the broadest enrichment across multiple compound classes, including ellagitannins (e.g., ellagic acid), phenolic acids (e.g., *p*-hydroxybenzoic, vanillic), flavonols (e.g., myricetin), stilbenes (e.g., resveratrol), and curcuminoids (e.g., curcumin).

UAE–acetone and UAE–ethanol clustered together, indicating a greater influence of the extraction method than solvent on overall composition. Acetone-based extracts were particularly rich in hydroxycinnamic acids (e.g., caffeic, ferulic) and flavonols (e.g., isorhamnetin, galangin), while 70% EtOH favored the recovery of simple phenols (e.g., pyrogallol), flavan-3-ols (e.g., catechin), and flavones (e.g., chrysin).

Soxhlet extracts, especially SE–acetone, were enriched in moderately polar constituents such as epicatechin, vitexin, sinapic, and chlorogenic acid.

**Figure 4:**
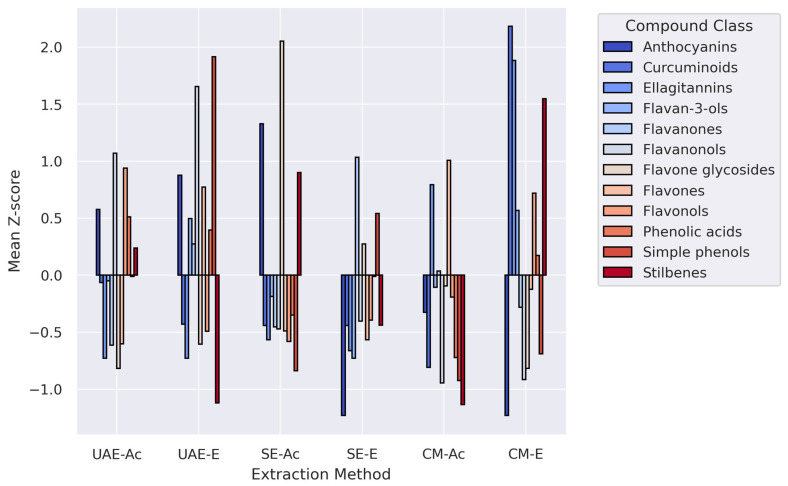
Mean z-score values of phenolic compound classes in *P. lentiscus* L. resin extracts obtained by different extraction methods—UAE-(Ac)etone, UAE-(E)thanol, SE-(Ac)etone, SE-(E)thanol, CM-(Ac)etone, and CM-(E)thanol. Each bar represents the average normalized abundance of all compounds within a given class for each extraction condition.

Average z-scores by phytochemical class (Figure 4) confirmed that CM–(E)tOH was the most chemically diverse extract, followed by UAE–(E)tOH. Among Soxhlet extracts, SE–(E)tOH showed more balanced recovery than SE–(Ac)etone. These findings underscore the combined impact of solvent polarity and extraction dynamics, with CM-EtOH emerging as the most comprehensive strategy for retrieving diverse phenolic metabolites from *P. lentiscus* L. resin.

The heatmap (Figure 3) and class-level summary (Figure 4) reflect distinct clustering patterns influenced primarily by extraction method, confirming the role of physical disruption (UAE), thermal gradient (SE), and passive diffusion (CM) in modulating extract composition.

### 3.3. Biological Activity

#### 3.3.1. DPPH and ABTS•^+^ Scavenging Activity

The antioxidant capacity of *P. lentiscus* L. resin extracts was evaluated towards DPPH and ABTS•^+^ free radicals over a concentration range of 0.01–0.20 mg/mL. Both assays showed comparable dose-dependent scavenging trends (Figures S2 and S3), with 70% EtOH extracts consistently exhibiting higher activity than acetone-based ones.

**Figure 5:**
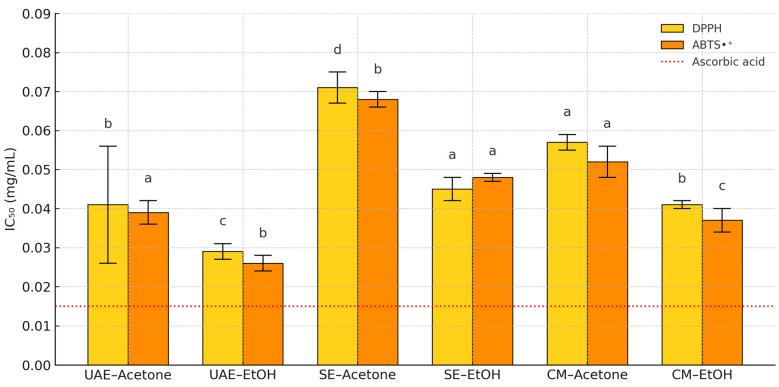
Antioxidant activity (IC_50_) of *P. lentiscus* L. resin extracts (UAE-acetone, UAE-EtOH, SE–acetone, SE-EtOH, CM–acetone, and CM-EtOH), evaluated by DPPH and ABTS•^+^ assays. Different letters above the bars indicate Tukey’s HSD groupings (α = 0.05); bars sharing a letter are not significantly different. The red dashed line represents the IC_50_ of ascorbic acid.

UAE–EtOH demonstrated the strongest activity, with IC_50_ values of 0.029 ± 0.002 mg/mL (DPPH) and 0.026 ± 0.002 mg/mL (ABTS•^+^), closely approaching that of the positive control, ascorbic acid (0.015 ± 0.001 mg/mL). SE–acetone and CM–acetone extracts showed moderate activity, while other combinations fell between these extremes. Comparative IC_50_ values and standard deviations are visualized in Figure 5, with full numerical data presented in Table S7.

Statistical analysis confirmed these observations. Two-way ANOVA (Table S8) revealed a significant effect of extraction condition on IC_50_ values (F = 147.64, *p* < 0.001), while no significant differences were observed between assay type or interaction terms, indicating consistent ranking across assays. Tukey’s post hoc analysis (Tables S9 and S10) confirmed that UAE–EtOH exhibited significantly greater antioxidant activity than all other extracts (*p* < 0.001).

These results highlight the synergistic interplay of UAE and EtOH in enhancing the radical scavenging potential of *P. lentiscus* L. resin.

#### 3.3.2. Contribution of Phenolic Compounds to the Radical Scavenging Activity

All compounds included in the correlation analyses were structurally confirmed and quantified as described in Section 3.3.1, ensuring that the correlations reflect experimentally validated phenolic–activity relationships. Pearson correlation analysis revealed significant negative associations (*p* < 0.05) between IC_50_ values and both TPC and TFC in DPPH (r = –0.803 and –0.806, respectively) and ABTS•^+^ assays (r = –0.740 and –0.732), indicating that higher total content is linked to stronger antioxidant activity.

**Figure 6:**
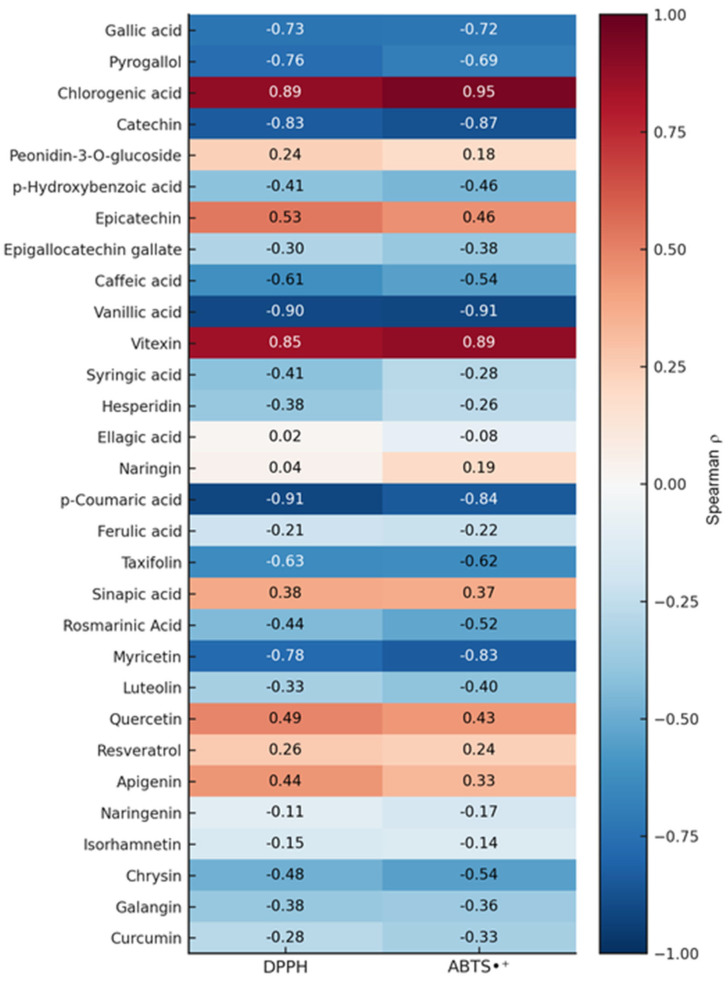
Spearman correlation heatmap of phenolic compounds from *P. lentiscus* L. resin extracts and their antioxidant activity (IC_50_ values) in DPPH and ABTS•^+^ assays. Negative correlations (blue) indicate higher antioxidant capacity.

To identify individual contributors, Spearman correlation analysis was performed using compound concentrations due to their non-normal distribution. Eleven phenolics showed significant correlations (*p* < 0.05) with IC_50_ values in both assays (Figure 6). Vanillic acid, *p*-coumaric acid, catechin, myricetin, pyrogallol, and gallic acid displayed strong inverse correlations (ρ ≤ –0.72), suggesting a key role in enhancing radical scavenging, whereas chlorogenic acid and vitexin were positively correlated with IC_50_ values, likely reflecting their abundance in less active extracts.

**Figure 7:**
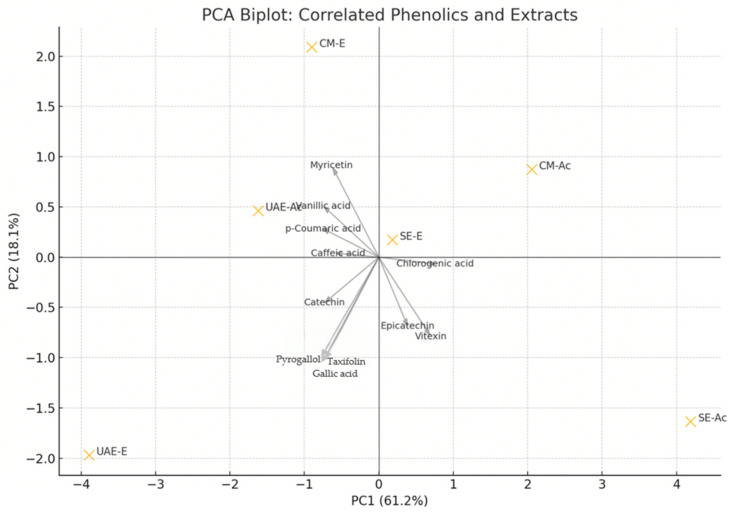
Principal component analysis (PCA) biplot of phenolic compounds and resin extracts of *P. lentiscus* L (UAE-(Ac)etone, UAE-(E)thanol, SE-(Ac)etone, SE-(E)thanol, CM-(Ac)etone, and CM-(E)thanol. The analysis is based on phenolics significantly correlated (|ρ| ≥ 0.5, *p* < 0.05) with antioxidant activity. UAE–EtOH is distinctly associated with gallic acid, pyrogallol, catechin, and taxifolin, indicating their major contribution to its activity.

Principal component analysis (PCA) based on phenolics with |ρ| ≥ 0.5 further supported these findings (Figure 7). UAE–EtOH was closely associated with gallic acid, pyrogallol, catechin, and taxifolin, aligning with its potent antioxidant profile. In contrast, SE–acetone and CM–acetone—characterized by lower activity—were positioned in the opposite quadrant. Hierarchical clustering based on the same key phenolics (Figure S4) confirmed the unique phenolic signature of UAE–EtOH, highlighting its compositional distinction and high radical scavenging potential, and separating it from less active extracts such as SE–acetone and CM–acetone, which were associated with higher chlorogenic acid and vitexin content.

It should be noted that phenolic concentrations were determined from single measurements per extract; thus, correlation, PCA, and clustering analyses are presented as exploratory tools to visualize potential associations between composition and antioxidant activity, rather than as inferential statistics.

#### 3.3.3. Antibacterial Activity

The resin extracts exhibited low to moderate antibacterial activity against both Gram-positive (*S. aureus*, *M. luteus*) and Gram-negative (*E. coli*, *P. aeruginosa*) strains (Table S11). Although inhibition zones were measurable, none of the extracts approached the activity of the reference antibiotic gentamicin.

The most pronounced effects were observed for SE–EtOH against *E. coli* (15.0 ± 0.2 mm) and CM–EtOH against *M. luteus* (12.3 ± 0.1 mm). However, overall differences among extraction conditions were relatively minor. No MIC or MBC could be established within the tested range (1.7–13.3%), indicating limited potency at the extract level.

These results suggest that the antibacterial effects of *P. lentiscus* L. resin extracts are modest and likely require further purification or concentration to reveal clinically relevant bioactivity.

#### 3.3.4. Antifungal Activity

The resin extracts of *P. lentiscus* L. demonstrated measurable inhibition against all tested fungal strains, with distinct sensitivity patterns (Table S12). Among them, the yeast *R. glutinis*, the filamentous fungus *A. niger*, and the mold-like *G. candidum* exhibited the highest responsiveness.

The yeast *R. glutinis* showed consistently strong susceptibility across most extracts, with inhibition zones ranging from 18.5 to 20.0 mm—closely matching the activity of the reference antifungal cycloheximide (20.5 ± 0.3 mm). MICs for this strain ranged from 1.7% to 13.3%, and MFC = 13.3% was recorded in multiple acetone- and EtOH-based extracts.

The fungus *G. candidum* exhibited its highest sensitivity to the SE–acetone extract (20.0 ± 0.1 mm, MIC = 1.7%), while *A. niger* responded most strongly to the SE–EtOH extract (20.0 ± 0.1 mm, MIC = 1.7%). These results highlight the influence of both extraction strategy and solvent polarity on antifungal efficacy.

Two-way ANOVA confirmed significant effects of extraction method, solvent, and their interaction (*p* < 0.001; Table S13). Post hoc Tukey comparisons (Table S14) further revealed that the SE–acetone extract was significantly more effective against *G. candidum* than UAE–acetone and CM–EtOH (*p* < 0.001). For *A. niger*, the SE–EtOH extract outperformed all others (*p* < 0.001), whereas *R. glutinis* showed relatively uniform inhibition across several extracts, with minor yet significant differences between CM–EtOH and UAE–EtOH.

Collectively, these findings underscore the pivotal role of optimized extraction conditions—particularly Soxhlet extraction using acetone or 70% EtOH—in enhancing the antifungal properties of *P. lentiscus* resin extracts.

#### 3.3.5. Contribution of Phenolic Compounds to the Antifungal Activity

Correlation analysis revealed no significant associations between TPC or TFC content and antifungal activity, suggesting that overall phenolic richness is not a reliable predictor of efficacy. Instead, individual phenolic compounds may play a more decisive role.

Spearman correlation analysis at the compound level identified four phenolics significantly associated with antifungal activity across the tested fungal strains (*p* < 0.05; Table S15). Vitexin exhibited a strong positive correlation (ρ = 0.986), followed by chrysin (ρ = 0.853). In contrast, myricetin (ρ = –0.899) and resveratrol (ρ = –0.833) showed negative correlations, suggesting potential antagonistic effects.

**Figure 8:**
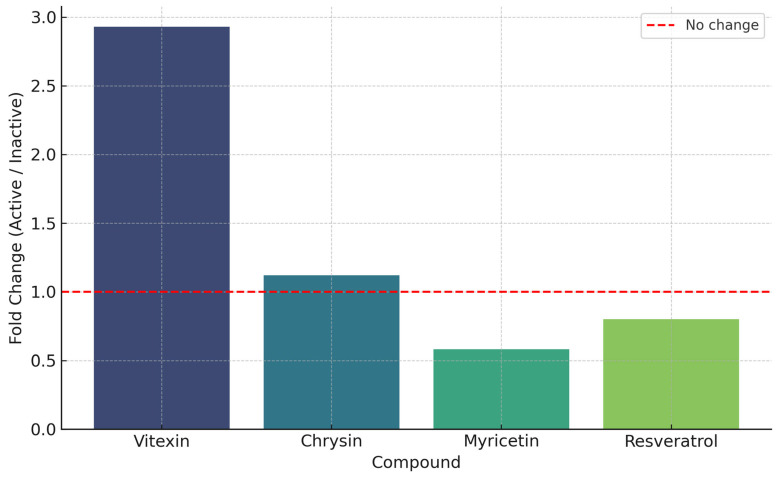
Fold change (active/inactive extracts) of key phenolic compounds in *P. lentiscus* L. resin extracts significantly correlated with antifungal activity. Compounds above the red line (fold change > 1) were more abundant in active extracts.

Both vitexin and chrysin were more abundant in the most active extracts, with fold changes of 2.93 and 1.12, respectively, compared to less active samples (Figure 8). Conversely, myricetin and resveratrol were relatively enriched in inactive extracts (fold change < 1), reinforcing their negative associations. Fold change values represent the ratio of compound abundance in active versus inactive extracts, with values >1 indicating enrichment in active samples.

These findings support the view that specific phenolic constituents, rather than total content, are key modulators of antifungal efficacy, depending on their relative abundance and extract composition.

#### 3.3.6. Cytotoxicity Evaluation

**Figure 9:**
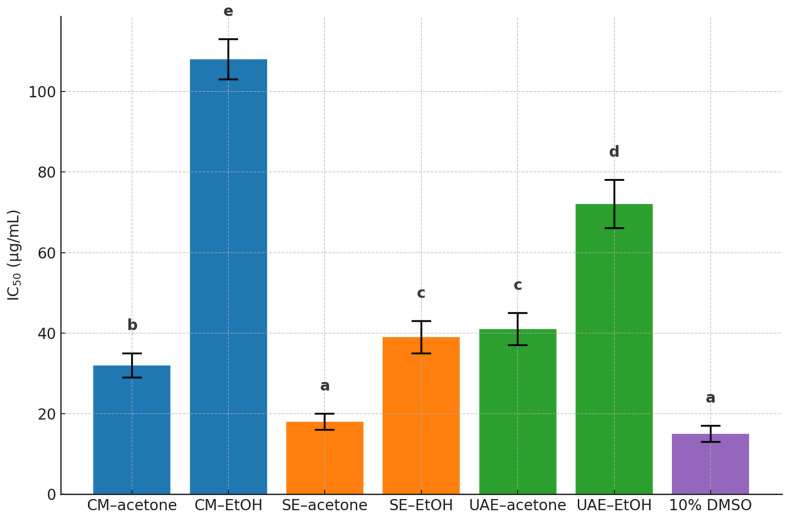
Cytotoxic activity (IC_50_ ± SD, µg/mL) of *P. lentiscus* L. resin extracts against MIA PaCa-2 cells. Extracts included CM-, SE-, and UAE-derived fractions with acetone or 70% EtOH, along with 10% DMSO as positive control. Different letters indicate significant differences (*p* < 0.05, two-way ANOVA with Tukey’s HSD test).

All *P. lentiscus* L. resin extracts exhibited dose-dependent cytotoxic effects against MIA PaCa-2 human pancreatic cancer cells, as determined by WST-8 assay following 24 h exposure (Figure S5). Among the tested samples, the SE–acetone extract demonstrated the strongest activity, with an IC_50_ value of 18.74 ± 1.62 µg/mL, followed by CM–acetone (32.65 ± 2.35 µg/mL) and SE–EtOH (39.28 ± 2.97 µg/mL) (Figure 9).

Extracts obtained via UAE showed moderate effects, with IC_50_ values of 40.79 ± 3.88 µg/mL (UAE–acetone) and 75.85 ± 5.43 µg/mL (UAE–EtOH). The CM–EtOH extract was the least active (IC_50_ = 108.74 ± 4.12 µg/mL).

Two-way ANOVA confirmed significant effects of extraction method, solvent type, and their interaction (*p* < 0.0001) (Table S16). Tukey’s post hoc analysis revealed that SE–acetone was significantly more potent than all other extracts (*p* < 0.001), whereas CM–EtOH was significantly less active (Table S17). No statistical difference was observed between SE–EtOH and UAE–acetone (*p* = 0.998), indicating comparable moderate activity.

Correlation analysis further identified chlorogenic acid (ρ = –0.886, *p* = 0.019) and vitexin (ρ = –0.771, *p* = 0.034) as significantly associated with cytotoxicity. Both compounds were particularly abundant in SE–acetone, the extract with the lowest IC_50_, suggesting a possible contribution to its antiproliferative effects. Whether these compounds act synergistically or independently remains to be elucidated through targeted mechanistic studies.

## 4. Discussion

### 4.1. Influence of Extraction Method and Solvent on Yield and Composition

All extractions were performed on defatted resin, a step that likely removed most lipid-rich terpenoid fraction and facilitated the recovery of low-abundance phenolics [24].

Extraction method strongly influenced yields, reflecting the underlying mass-transfer processes. UAE achieved the highest recoveries, particularly with acetone (>80%), due to acoustic cavitation and matrix disruption [34,35,36]. SE, although exhaustive, relies on prolonged reflux; heating can compromise thermolabile compounds, explaining its intermediate yields and compositional shifts [37,38,39,40]. CM, limited to passive diffusion at ambient temperature, consistently produced the lowest yields [39,41].

Solvent effects depended on both polarity and proticity. Acetone (polar aprotic, intermediate polarity) efficiently solubilized semi-polar phenolic acids and aglycone-rich flavonoids, whereas 70% ethanol (polar protic) recovered more hydrophilic subclasses, aided by water-driven matrix hydration. Sequential application of acetone followed by ethanol generated compositionally distinct yet complementary extracts, as shown in the clustered heatmap (Figure 3 and Figure 4; Table S6) [37,42].

Targeted UHPLC-ESI-MS/MS confirmed these polarity-driven trends. UAE–acetone extracts were enriched in hydroxycinnamic acids and flavonols (e.g., isorhamnetin), while UAE–EtOH favored simple phenols and phenolic esters (e.g., rosmarinic and chlorogenic acids). CM recovered less polar flavonoids such as naringenin in acetone, and higher ellagic acid in 70% EtOH. SE–acetone was characterized by flavan-3-ols and C-glycosyl flavones, whereas SE–EtOH displayed a more hydrophilic profile enriched in benzoic acid derivatives and glycosylated flavanones. Overall, extraction conditions critically shaped the resin’s phenolic signature [43,44,45]. These compositional patterns provide the foundation for understanding how extraction conditions translate into biological activities, as explored in the next section.

### 4.2. Relationship Between Composition and Biological Activity

The differences in phenolic composition described above were directly reflected in the antioxidant, antifungal, and cytotoxic responses of the extracts. Two-way ANOVA confirmed that these biological activities were significantly influenced by extraction parameters (*p* < 0.001), underscoring the importance of method and solvent in modulating efficacy [5,45,46,47].

The antioxidant capacity of the extracts, assessed by DPPH and ABTS•^+^ assays, correlated strongly with phenolic and flavonoid contents. UAE–EtOH showed the greatest activity, with IC_50_ values comparable to ascorbic acid, consistent with its enrichment in gallic acid, pyrogallol, catechin, and taxifolin—compounds well established as radical scavengers [48,49,50,51]. In contrast, less active extracts such as SE–acetone and CM–acetone were characterized by chlorogenic acid and vitexin as major constituents. Multivariate analyses (PCA and hierarchical clustering) further supported these associations, indicating that both individual compounds and synergistic interactions contribute to activity patterns. These trends should be interpreted as indicative rather than strictly causal, given the reliance on single-concentration data.

Antibacterial activity was generally low to moderate, whereas antifungal responses were more pronounced and selectively modulated by extraction. SE–acetone, SE–EtOH, and UAE–EtOH extracts exhibited the strongest inhibition against *G. candidum*, *A. niger*, and *R. glutinis*, respectively. No correlation with bulk TPC or TFC was observed; instead, activity was linked to specific phenolics, particularly vitexin and chrysin. These flavones have been associated with distinct antifungal mechanisms: vitexin with plasma membrane disruption and ergosterol biosynthesis interference, and chrysin with inhibition of fungal efflux pumps, leading to intracellular accumulation of toxic compounds [50,51,52,53,54]. Such compound-specific actions highlight the importance of targeted metabolite enrichment over total phenolic measures [55].

Cytotoxicity against MIA PaCa-2 cells was highest in the SE–acetone extract, which was enriched in chlorogenic acid and vitexin—both previously associated with antiproliferative effects, especially in pancreatic and breast cancer cell lines [56,57]. Their abundance likely underpins the stronger cytotoxic response observed in this extract.

Collectively, these findings emphasize that bioactivity is more reliably predicted by the selective enrichment of key phenolics—shaped by extraction strategy—than by total phenolic levels alone [49,58,59]. This highlights the potential of strategically optimized extractions to yield resin-derived products with tailored properties. Importantly, these compound-specific associations indicate that extraction design is not only a chemical determinant but also a strategic tool, as further discussed in the context of extraction optimization below.

### 4.3. Strategic Considerations for Extraction Optimization

Building on the composition–activity relationships, the following section discusses how these findings can inform practical extraction strategies. The comparative analysis demonstrates that both extraction method and solvent polarity should be selected in alignment with the intended biological target. Based on the experimental results, optimal combinations can be identified for each bioactivity type:

- Antioxidant applications (nutraceuticals, cosmetics): UAE–EtOH is the most suitable strategy, yielding high levels of electron-donating phenolics with strong radical-scavenging activity. The method also maintains eco-efficiency and minimizes thermal degradation.
- Antifungal applications: SE–acetone and SE–EtOH provided enhanced efficacy against *G. candidum* and *A. niger*, respectively, consistent with the enrichment of moderately polar compounds such as vitexin and chrysin. UAE–EtOH was effective against *R. glutinis*, offering a greener alternative with comparable results.
- Cytotoxic applications (anticancer research): SE–acetone exhibited the highest potency against MIA PaCa-2 cells, consistent with its enrichment in chlorogenic acid and vitexin. This method appears particularly effective for recovering phenolics that are strongly matrix-associated or less accessible to milder solvents.

From an environmental and industrial standpoint, UAE uses shorter cycles and a lower thermal load than Soxhlet, reducing both energy demand and solvent losses [60,61]. Acetone and 70% ethanol are readily recoverable via rotary evaporation or closed-loop capture, while ethanol additionally aligns with green-extraction principles [62]. UAE is also scalable (probe- or flow-through systems), whereas Soxhlet is batch-limited and rarely employed beyond laboratory scale [63,64].

Taken together, these operational considerations complement the bioactivity-led solvent choices above. A strategy-specific approach, guided by targeted chemical profiling and bioassays, can maximize the therapeutic potential of resin-derived extracts while maintaining process eco-efficiency [36,38]. This is aligned with current green-extraction trends in the pharmaceutical, food, and cosmetic sectors [52].

These operational and application-oriented insights provide a practical framework, which, when placed in the context of earlier studies, highlights the novelty and added value of the present work.

### 4.4. Novelty of This Study in Context of Previous Research

This work presents the first systematic phenolic profiling of Moroccan *P. lentiscus* L. resin, with 30 compounds identified and quantified across extracts. All detected constituents are reported here for the first time in Moroccan mastic gum. Several—chlorogenic, caffeic, rosmarinic, and ellagic acids, as well as taxifolin, luteolin, quercetin, apigenin, naringenin, and chrysin—have previously been described in resin of Turkish origin [8]. Notably, ellagic acid, rosmarinic acid, and isorhamnetin emerged as dominant constituents in Moroccan samples, suggesting compositional distinctions that may reflect geographic or genetic variation within *P. lentiscus* L. These findings highlight the importance of regional characterization for uncovering overlooked metabolites and clarifying their role in bioactivity.

This study advances the field in several key ways:

- Defatting-enabled enrichment—facilitated recovery of low-abundance phenolics.
- Polarity–proticity-guided sequential extraction—produced distinct and complementary profiles.
- Compound-specific bioactivity associations—linked activity to gallic acid, vitexin, and others.
- Bioactivity-driven extraction strategies—identified optimal solvent–method combinations.

Altogether, Moroccan mastic gum is established as a chemically diverse and functionally versatile source of non-volatile phenolics. Beyond expanding the phytochemical record, the methodological framework developed here demonstrates how extraction design can be strategically tailored to maximize biological potential—an approach not previously illustrated for *Pistacia* resins.

While these advances represent significant progress in characterizing Moroccan *P. lentiscus* L. resin, it is equally important to recognize the study’s boundaries. A transparent appraisal of limitations is necessary to contextualize the findings and guide future work.

### 4.5. Limitations and Perspectives for Future Research

First, extraction yields were obtained from single determinations (n = 1) due to limited resin availability; thus, yield values should be considered indicative only. Harvesting *P. lentiscus* L. resin is seasonal and labor-intensive, which restricted replication in this study and limited the possibility of performing additional bioassays. Future work will address this by scaling up collections and performing replicated extractions for statistical validation.

Second, the chemical profiling employed a targeted UHPLC–ESI–MS/MS panel of 30 phenolic compounds. Due to the targeted nature of this approach, additional phenolics—such as glycosylated derivatives or high-molecular-weight compounds—may have been overlooked. To overcome this, complementary untargeted profiling and integrative omics strategies will be needed to provide a more complete chemical picture [65].

Third, correlations observed with antioxidant, antifungal, and cytotoxic activities should be interpreted as exploratory and require mechanistic validation. Future investigations will therefore focus on testing purified compounds, spiking experiments, and bioactivity-guided fractionation in order to establish causal links between specific phenolics and biological outcomes.

In addition, the antioxidant assessment was limited to DPPH and ABTS assays, which are internationally recognized for comparative screening but do not capture the full complexity of oxidative processes. Expanding the evaluation to include additional in vitro assays (e.g., FRAP, ORAC) and cellular models of oxidative stress will provide a more comprehensive and biologically relevant perspective.

The cytotoxicity evaluation was also limited in scope, as only one cancer cell line was tested, and the originally planned assessment on fibroblast cells was not carried out. This omission constrains conclusions regarding the general cytotoxicity of the extracts toward non-cancerous human cells. Future work should therefore incorporate normal cell lines, additional cancer models, in vivo assays, and pathway-level analyses to enhance translational relevance.

Finally, although Soxhlet and UAE yielded promising extracts, their scalability and environmental footprint warrant further assessment. Comparative studies incorporating green-extraction metrics and process optimization will be valuable for assessing industrial feasibility.

Taken together, addressing these limitations in future work will not only refine mechanistic understanding but also strengthen the translational potential of Moroccan *P. lentiscus* L. resin.

## 5. Conclusions

This study provides the first comprehensive, compound-level phenolic profile of Moroccan *P. lentiscus* L. resin, identifying and quantifying 30 constituents across multiple structural classes, most reported here for the first time in mastic gum. A preparatory defatting step enhanced phenolic recovery by reducing interference from the resin’s abundant terpenoids. Comparative application of three extraction methods (cold maceration, Soxhlet, ultrasound-assisted extraction) within a polarity–proticity-guided sequential scheme (acetone → 70% ethanol) showed that solvent properties and extraction dynamics jointly shape phenolic subclass composition. The resulting extracts displayed distinct chemical signatures that translated into differentiated bioactivity profiles. Composition–activity correlations indicated that gallic acid, pyrogallol, catechin, and taxifolin were linked to antioxidant effects; vitexin and chrysin to antifungal activity; and chlorogenic acid and vitexin to cytotoxicity. Selective enrichment of key phenolics was a stronger predictor of bioactivity than total phenolic content. Overall, UAE–EtOH proved optimal for antioxidant applications, SE–acetone or SE–EtOH for antifungal applications, and SE–acetone for cytotoxic activity. By integrating compositional, functional, and methodological insights, Moroccan mastic gum emerges as a chemically diverse and functionally versatile source of non-volatile phenolics with significant potential for tailored nutraceutical, pharmaceutical, and cosmetic applications.

## Data Availability

The original contributions presented in this study are included in the article. Further inquiries can be directed to the corresponding authors.

## Supplementary Materials

The following supporting information can be downloaded at: https://www.mdpi.com/article/10.3390/antiox14101207/s1, Figure S1: Chromatographic profiles of *P. lentiscus* L. resin extracts analyzed by UHPLC-ESI-MS/MS; Figure S2: Dose-dependent DPPH radical scavenging activity (% inhibition) of *P. lentiscus* L. resin extracts obtained by CM (a), SE (b), and UAE (c) using acetone and EtOH sequentially as extraction solvents. Ascorbic acid was used as a reference antioxidant; Figure S3: Dose-dependent ABTS•^+^ radical scavenging activity of *P. lentiscus* L. resin extracts obtained by CM (a), SE (b), and UAE (c) using acetone and EtOH sequentially as extraction solvents. Ascorbic acid was used as a reference antioxidant; Figure S4: Hierarchical clustering dendrogram of *P. lentiscus* L. resin extracts based on four key phenolics (Euclidean distance, Ward’s method). Clustering was based on the concentrations of gallic acid, pyrogallol, catechin, and taxifolin—identified as top contributors to antioxidant activity. UAE–EtOH formed a distinct cluster, highlighting its unique phenolic signature. Figure S5: Dose–response cytotoxicity curves of *Pistacia lentiscus* L. resin extracts obtained by CM, SE, and UAE, using acetone and ethanol as solvents. Cell viability was assessed after 24 h treatment of MIA PaCa-2 cells via WST-8 assay. Data are expressed as mean ± SD (n = 3). Concentration range: 50–500 µg/mL; Table S1: Total phenolic content (TPC) and total flavonoid content (TFC) of *P. lentiscus* L. resin extracts, obtained by ultrasound-assisted extraction (UAE), Soxhlet extraction (SE), and cold maceration (CM) and with acetone and ethanol (EtOH) applied as sequent solvents. Data represent the mean from three measurements ± standard deviation; Table S2: Two-way ANOVA results for TPC of *P. lentiscus* L. resin extracts obtained by different combinations of extraction methods (UAE, SE, and CM) and solvents (acetone and EtOH); Table S3: Two-way ANOVA results for TFC of *P. lentiscus* L. resin extracts obtained by different combinations of extraction methods (UAE, SE, and CM) and solvents (acetone and EtOH); Table S4: Tukey’s post hoc test results for TPC of *P. lentiscus* L. resin extracts obtained by different combinations of extraction methods (UAE, SE, and CM) and solvents (acetone and EtOH); Table S5: Tukey’s post hoc test results for TFC of *P. lentiscus* L. resin extracts obtained by different combinations of extraction methods (UAE, SE, and CM) and solvents (acetone and EtOH); Table S6: Identification, calibration data, and concentrations of phenolic compounds in *P. lentiscus* L. resin extracts. The table presents all compounds identified and quantified by UHPLC-ESI-MS/MS in resin extracts obtained through UAE, SE, and CM using acetone (Ac) and ethanol (E) as solvents. For each compound, the retention time (RT), calibration equation, and correlation coefficient (R^2^) are provided, along with concentration values determined under each extraction condition. N.d. = not detected; Table S7: IC_50_ values (mg/mL) for DPPH and ABTS•^+^ radical scavenging activity of *P. lentiscus* L. resin extracts obtained by CM, SE, and UAE using acetone and EtOH as solvents; Table S8: Two-way ANOVA summary for the effects of extraction condition and assay type on IC_50_ values obtained from DPPH and ABTS•^+^ radical scavenging tests of the *P. lentiscus* L. resin extracts; Table S9: Tukey HSD post hoc comparison of IC_50_ values among extraction conditions in the DPPH assay; Table S10: Tukey HSD post hoc comparison of IC_50_ values among extraction conditions in the ABTS•^+^ assay; Table S11: Antibacterial activity of *P. lentiscus* L. resin extracts obtained by CM, SE, and UAE, sequentially using acetone and EtOH as solvents. Inhibition zone diameters (mm) and minimum inhibitory/bactericidal concentrations (MIC/MBC, %) against selected bacterial strains; Table S12: Antifungal activity of *P. lentiscus* L. resin extracts obtained by CM, SE, and UAE, each performed sequentially using acetone and EtOH as solvents. Inhibition zone diameters (mm) and minimum inhibitory/fungicidal concentrations (MIC/MFC, %) against selected fungal strains; Table S13: Two-way ANOVA results for the antifungal activity of *P. lentiscus* L. resin extracts against *G. candidum*, *A. niger*, and *R. glutinis* strains. The analysis evaluates the effects of extraction method and solvent on inhibition zones (mm) for each strain; Table S14: Tukey HSD pairwise comparisons of inhibition zones (mm) for *G. candidum*, *A. niger*, and *R. glutinis* treated with *P. lentiscus* L. resin extracts; Table S15: Spearman correlation coefficients (ρ) between selected phenolic compounds of *P. lentiscus* L. resin extracts and antifungal activity (inhibition zone, mm) against three fungal strains; Table S16: Two-way ANOVA results for the cytotoxic activity of *P. lentiscus* L. resin extracts against MIA PaCa-2 human pancreatic cancer cell. The analysis evaluates the effects of extraction method and solvent on inhibition on IC_50_ (µg/mL) for each strain; Table S17: Tukey HSD pairwise comparisons of cytotoxicity (IC_50_, mg/mL) of *P. lentiscus* L. resin extracts against MIA PaCa-2 human pancreatic cancer cell.

## Abbreviations

The following abbreviations are used in this manuscript:

UAE	Ultrasound-Assisted Extraction
SE	Soxhlet Extraction
CM	Cold Maceration
TPC	Total Phenolic Content
TFC	Total Flavonoid Content
UHPLC–ESI–MS/MS	Ultra-High-Performance Liquid Chromatography Coupled with Electrospray Ionization Tandem Mass Spectrometry
DPPH	2,2-Diphenyl-1-picrylhydrazyl
ABTS•^+^	2,2′-Azino-bis(3-ethylbenzothiazoline-6-sulfonic acid
PCA	Principal Component Analysis
