# Supplementary material for: Extraction Methods Shape the Phenolic Composition and Bioactivities of Defatted Moroccan Pistacia lentiscus L. Resin
Source: Antioxidants (Basel). 2025 Oct 5;14(10):1207. doi: 10.3390/antiox14101207 (PMC12561634; doi:10.3390/antiox14101207)
Supplement: Supplementary file 1 [file antioxidants-14-01207-s001.zip › antioxidants-3885749-supplementary.pdf]

Table S1. Total phenolic content (TPC) and total flavonoid content (TFC) of *P. lentiscus* L. resin extracts, obtained by ultrasound-assisted extraction (UAE), Soxhlet extraction (SE), and cold maceration (CM) and with acetone and 70% ethanol (70% EtOH) applied as sequent solvents. Data represent the mean from three measurements ± standard deviation.

Extracts	TPC (mg GAE/g extract)	TFC (mg QE/g extract)
UAE-Acetone	49.4 ± 0.15	21.38 ± 0.34
UAE-EtOH	26.8 ± 0.20	13.22 ± 0.21
SE-Acetone	29.72 ± 0.30	18.78 ± 0.44
SE-EtOH	16.8 ± 0.12	11.53 ± 0.21
CM-Acetone	35.52 ± 0.25	15.8 ± 0.30
CM-EtOH	21.1 ± 0.15	8.95 ± 0.12

Table S2. Two-way ANOVA results for TPC of *P. lentiscus* L. resin extracts obtained by different combinations of extraction methods (UAE, SE, and CM) and solvents (acetone and 70% EtOH).

Source	sum_sq	df	F	PR(>F)
C(Method)	683.16	2	8086.41	< 0.0001
C(Solvent)	1247.48	1	29532.13	< 0.0001
C(Method):C(Solvent)	83.14	2	984.09	< 0.0001
Residual	0.51	12		

Table S3. Two-way ANOVA results for TFC of *P. lentiscus* L. resin extracts obtained by different combinations of extraction methods (UAE, SE, and CM) and solvents (acetone and 70% EtOH).

Source	sum_sq	df	F	PR(>F)
C(Method)	72.09	2	592.29	< 0.0001
C(Solvent)	244.09	1	4010.91	< 0.0001
C(Method):C(Solvent)	1.39	2	11.44	0.0017
Residual	0.73	12		

Table S4. Tukey's post hoc test results for TPC of *P. lentiscus* L. resin extracts obtained by obtained by different combinations of extraction methods (UAE, SE, and CM) and solvents (acetone and 70% EtOH).

Comparison	Mean Diff. (mg GAE/g extract)	*p*-value	Significant
CM-Acetone vs CM-EtOH	-14.42	< 0.001	Yes
CM-Acetone vs SE-Acetone	-5.8	< 0.001	Yes
CM-Acetone vs SE-EtOH	18.72	< 0.001	Yes
CM-Acetone vs UAE-Acetone	-13.88	< 0.001	Yes
CM-Acetone vs UAE-EtOH	8.62	< 0.001	Yes
CM-EtOH vs SE-Acetone	8.62	< 0.001	Yes
CM-EtOH vs SE-EtOH	33.14	< 0.001	Yes
CM-EtOH vs UAE-Acetone	-27.3	< 0.001	Yes
CM-EtOH vs UAE-EtOH	-5.8	< 0.001	Yes
SE-Acetone vs SE-EtOH	-12.92	< 0.001	Yes
SE-Acetone vs UAE-Acetone	19.88	< 0.001	Yes
SE-Acetone vs UAE-EtOH	2.82	< 0.001	Yes
SE-EtOH vs UAE-Acetone	-32.8	< 0.001	Yes
SE-EtOH vs UAE-EtOH	-11.1	< 0.001	Yes
UAE-Acetone vs UAE-EtOH	21.6	< 0.001	Yes

Table S5. Tukey's post hoc test results for TFC of *P. lentiscus* L. resin extracts obtained by different combinations of extraction methods (UAE, SE, and CM) and solvents (acetone and 70% EtOH).

Comparison	Mean Diff. (mg QE/g extract)	*p*-value	Significant
CM-Acetone vs CM-EtOH	-6.85	< 0.001	Yes
CM-Acetone vs SE-Acetone	2.98	< 0.001	Yes
CM-Acetone vs SE-EtOH	-4.27	< 0.001	Yes
CM-Acetone vs UAE-Acetone	5.58	< 0.001	Yes
CM-Acetone vs UAE-EtOH	-2.58	< 0.001	Yes
CM-EtOH vs SE-Acetone	-3.87	< 0.001	Yes
CM-EtOH vs SE-EtOH	2.58	< 0.001	Yes
CM-EtOH vs UAE-Acetone	12.43	< 0.001	Yes
CM-EtOH vs UAE-EtOH	4.27	< 0.001	Yes
SE-Acetone vs SE-EtOH	-7.25	< 0.001	Yes
SE-Acetone vs UAE-Acetone	9.45	< 0.001	Yes
SE-Acetone vs UAE-EtOH	1.28	< 0.001	Yes
SE-EtOH vs UAE-Acetone	-16.7	< 0.001	Yes
SE-EtOH vs UAE-EtOH	-5.97	< 0.001	Yes
UAE-Acetone vs UAE-EtOH	10.72	< 0.001	Yes

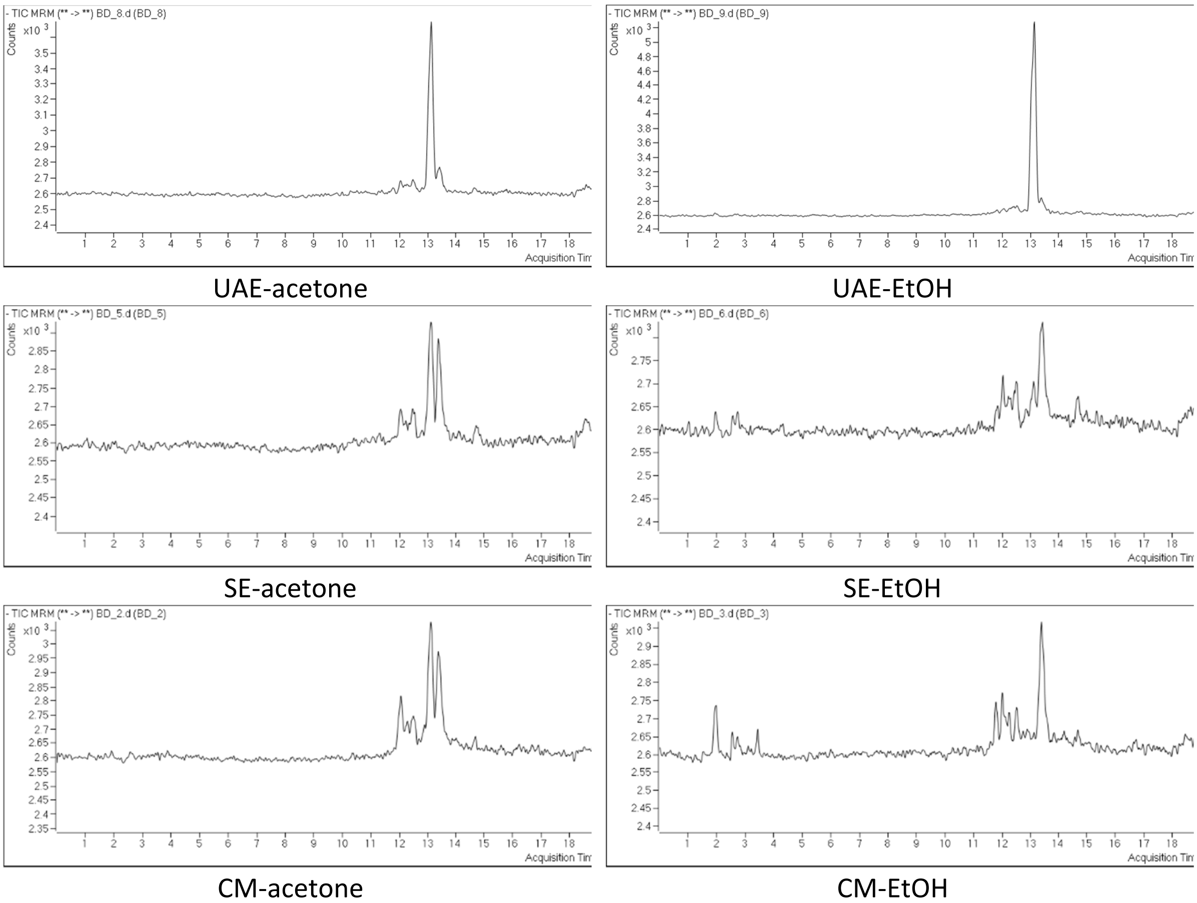

Figure S1. Chromatographic profiles of *P. lentiscus* L. resin extracts analyzed by UHPLC-ESI-MS/MS.

Table S6. Identification, calibration data, and concentrations of phenolic compounds in *P. lentiscus* L. resin extracts. The table presents all compounds identified and quantified by UHPLC-ESI-MS/MS in resin extracts obtained through UAE, SE, and CM using acetone (Ac) and 70% ethanol (E) as solvents. For each compound, the retention time (RT), calibration equation, and correlation coefficient (R^2) are provided, along with concentration values determined under each extraction condition. N.d. = not detected.

**Compounds**	**Equation**	**R²**	**R.T (min)**	**UAE**		**SE**		**CM**	
				**Concentration (µg/g extract)**					
				**Ac**	**E**	**Ac**	**E**	**Ac**	**E**
Gallic acid	y=29.1072x+448.85	0.9995	5.58	1.36	3.12	0.45	0.93	0.37	0.42
Pyrogallol	y=0.8931x+27.15	0.9993	6.71	0.94	1.85	0.55	1.2	0.51	0.62
Chlorogenic acid	y=26.3508x+815.02	0.9994	10.77	1.02	0.42	1.49	1.16	1.05	0.74
Catechin	y=8.766x+278.55	0.9993	10.92	0.88	2.63	0.33	0.69	0.58	1.34
Peonidin-3-*O*-glucoside	y=4.986x+9.393	0.9996	10.99	0.13	0.15	0.18	0.01	0.07	0.01
p-Hydroxybenzoic acid	y=14.985x+1091.18	0.9968	11.25	2.35	3.14	1.88	2.68	2.51	6.69
Epicatechin	y=11.252x+458.68	0.9985	11.36	1.23	2.04	3.51	1.12	1.41	2.25
Epigallocatechin gallate	y=21.589x+100.43	0.9994	11.52	0.36	0.18	0.09	0.18	0.36	0.36
Caffeic acid	y=93.695x+6274.49	0.9921	11.58	6.54	3.60	1.14	3.65	1.25	2.18
Vanillic acid	y=0.485x+22.154	0.9984	11.59	1.36	1.69	0.34	1.15	0.64	1.92
Vitexin	y=70.596x+3664.04	0.998	11.66	0.32	0.51	2.90	1.3	0.97	0.32
Syringic acid	y=0.7241x+16.594	0.999	11.66	1.39	0.29	n.d.	1.56	0.43	0.81
Hesperidin	y=11.326x+308.005	0.9994	12.02	0.20	0.41	0.18	0.66	0.35	0.35
Ellagic acid	y=2.586x+263.52	0.9956	12.04	0.12	0.15	2.96	1.28	26.8	45.88
Naringin	y=13.341x+250.588	0.9998	12.22	0.17	0.17	0.42	0.85	0.09	0.58
p-Coumaric acid	y=50.281x+1892.54	0.9991	12.30	3.60	3.71	0.41	3.34	0.50	3.60
Ferulic acid	y=4.352x+128.453	0.9995	12.40	2.80	1.63	1.63	0.42	0	0.85
Taxifolin	y=41.639x+4556.76	0.9939	12.43	4.32	5.49	1.23	1.37	0.29	0.35
Sinapic acid	y=4.351x + 42.737	0.9994	12.49	0	0.52	0.87	0.37	0.19	0.55
Rosmarinic Acid	y=4.065x - 49.228	0.9998	12.51	21.3	24.39	14.16	8.21	14.71	11.30
Myricetin	y=20.491x+1224.36	0.9978	12.77	3.00	3.00	0.59	1.88	2.15	4.23
Luteolin	y=117.40x+6198.38	0.9846	13.34	1.80	3.96	2.71	2.88	3.22	3.72
Quercetin	y=41.436x+2291.24	0.9926	13.46	1.01	0.38	1.50	1.38	2.50	2.28
Resveratrol	y=4.727x+106.32	0.9956	13.90	1.31	0.45	1.73	0.88	0.44	2.14
Apigenin	y=-0.0324x2+129.30x+3510.35	0.9983	13.94	0.52	0.29	0.63	0.27	2.10	0.63
Naringenin	y=47.851x+3816.28	0.9894	13.98	4.41	8.00	3.53	3.10	7.90	0.44
Isorhamnetin	y=-0.066x2+286.51x+11633.27	0.9966	14.11	23.2	1.00	2.80	1.20	1.61	1.32
Chrysin	y=8.365x+4646.058	0.998	15.34	2.52	4.72	0.81	0.97	2.97	0.32
Galangin	y=-0.0054x2+20.044x+896.523	0.9987	15.72	4.43	1.50	1.50	1.45	0	3.00
Curcumin	y=30.077x+8117.86	0.9989	16.19	0.67	0.34	0.33	0.33	0	2.70

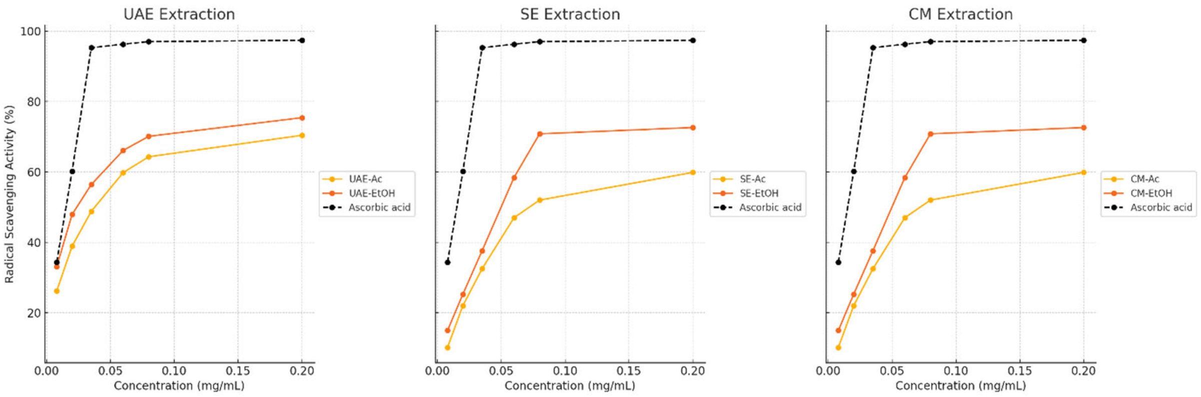

Figure S2. Dose-dependent DPPH radical scavenging activity (% inhibition) of *P. lentiscus* L. resin extracts obtained by CM (a), SE (b), and UAE (c) using acetone and 70% EtOH sequentially as extraction solvents. Ascorbic acid was used as a reference antioxidant.

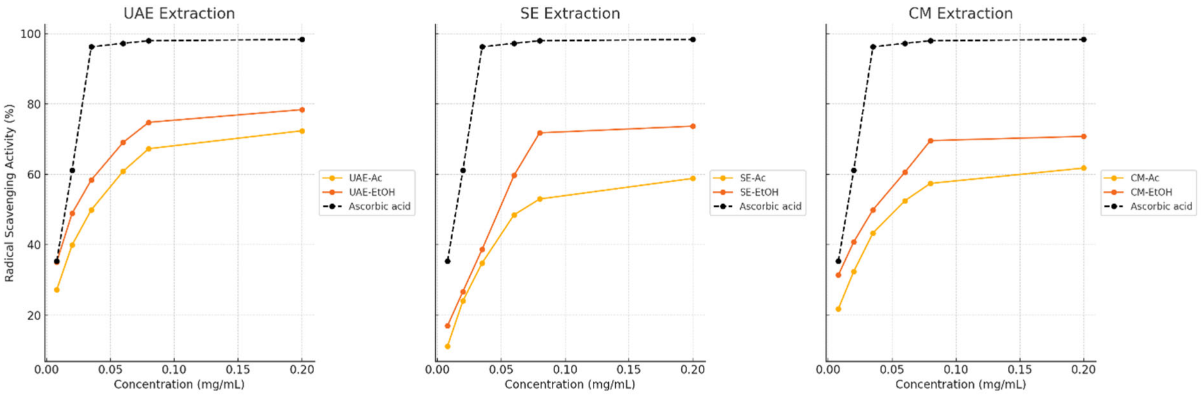

Figure S3. Dose-dependent ABTS•$^+$ radical scavenging activity of *P. lentiscus* L. resin extracts obtained by CM (a), SE (b), and UAE (c) using acetone and 70% EtOH sequentially as extraction solvents. Ascorbic acid was used as a reference antioxidant.

Table S7. IC_{50} values (mg/mL) for DPPH and ABTS•$^+$ radical scavenging activity of *P. lentiscus* L. resin extracts obtained by CM, SE, and UAE using acetone and 70% EtOH as solvents.

**Extraction solvent**	**UAE**		**SE**		**CM**	
	DPPH	ABTS•$^+$	DPPH	ABTS•$^+$	DPPH	ABTS•$^+$
Acetone	0.041 ± 0.015	0.039 ± 0.003	0.071 ± 0.004	0.068 ± 0.002	0.057 ± 0.002	0.052 ± 0.004
70% EtOH	0.029 ± 0.002	0.026 ± 0.002	0.045 ± 0.003	0.048 ± 0.001	0.041 ± 0.001	0.037 ± 0.003
Ascorbic acid	0.015 ± 0.001					

Note: All values are expressed as mean ± SD from three independent experiments (n = 3).

Table S8. Two-way ANOVA summary for the effects of extraction condition and assay type on IC_{50} values obtained from DPPH and $ABTS^{\bullet+}$ radical scavenging tests of the *P. lentiscus* L. resin extracts.

Source	df	sum_sq	mean_sq	F	PR(>F)
C(Assay)	0	0			
C(Extract)	5	0.006262	0.001252	147.6365	3.49E-20
C(Assay):C(Extract)	0	0			
Residual	30	0.000254	8.48E-06		

Table S9. Tukey HSD post hoc comparison of IC_{50} values among extraction conditions in the DPPH assay.

Comparison	Mean Diff. (mg/mL)	*p*-value	Significant
CM-Ac vs CM-E	-0.014	0.0002	TRUE
CM-Ac vs SE-Ac	0.017	0	TRUE
CM-Ac vs SE-E	-0.003	0.7039	FALSE
CM-Ac vs UAE-Ac	-0.012	0.001	TRUE
CM-Ac vs UAE-E	-0.025	0	TRUE
CM-E vs SE-Ac	0.031	0	TRUE
CM-E vs SE-E	0.011	0.0021	TRUE
CM-E vs UAE-Ac	0.002	0.9219	FALSE
CM-E vs UAE-E	-0.011	0.0021	TRUE
SE-Ac vs SE-E	-0.02	0	TRUE
SE-Ac vs UAE-Ac	-0.029	0	TRUE
SE-Ac vs UAE-E	-0.042	0	TRUE
SE-E vs UAE-Ac	-0.009	0.0098	TRUE
SE-E vs UAE-E	-0.022	0	TRUE
UAE-Ac vs UAE-E	-0.013	0.0005	TRUE

Table S10. Tukey HSD post hoc comparison of IC_{50} values among extraction conditions in the $ABTS^{\bullet+}$ assay.

Comparison	Mean Diff. (mg/mL)	*p*-value	Significant
CM-Ac vs CM-E	-0.012	0.0001	TRUE
CM-Ac vs SE-Ac	-0.018	0	TRUE
CM-Ac vs SE-E	-0.002	0.8551	FALSE
CM-Ac vs UAE-Ac	0.001	0.9482	FALSE
CM-Ac vs UAE-E	-0.019	0	TRUE
CM-E vs SE-Ac	-0.006	0.0243	TRUE
CM-E vs SE-E	0.01	0.0001	TRUE
CM-E vs UAE-Ac	0.013	0	TRUE
CM-E vs UAE-E	-0.007	0.0127	TRUE
SE-Ac vs SE-E	0.016	0	TRUE
SE-Ac vs UAE-Ac	0.019	0	TRUE
SE-Ac vs UAE-E	-0.001	0.9555	FALSE
SE-E vs UAE-Ac	0.003	0.5583	FALSE
SE-E vs UAE-E	-0.017	0	TRUE
UAE-Ac vs UAE-E	-0.02	0	TRUE

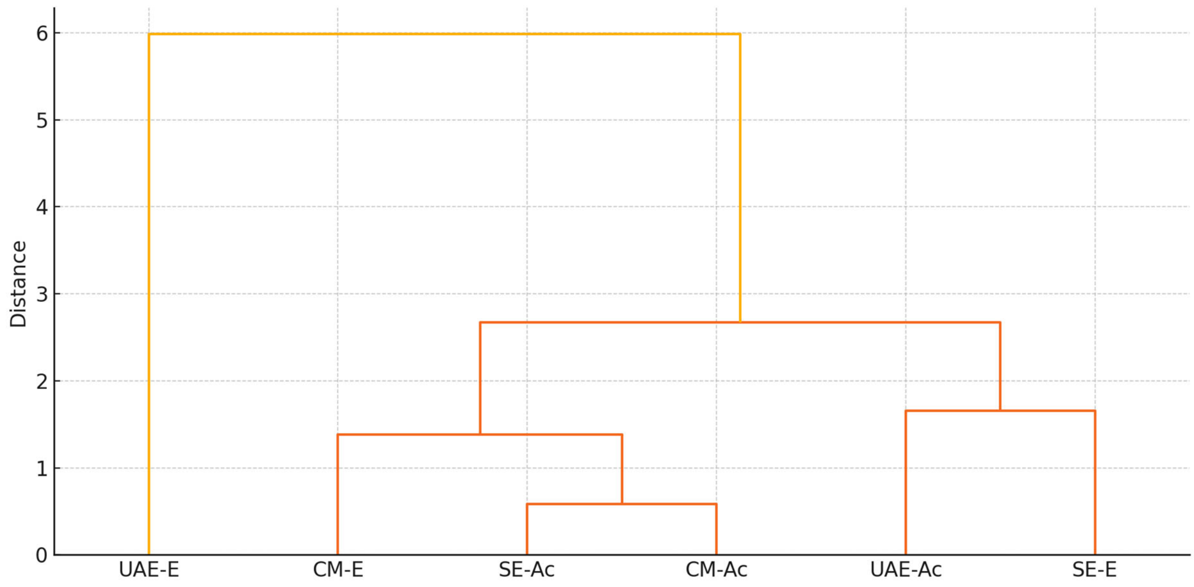

Figure S4. Hierarchical clustering dendrogram of *P. lentiscus* L. resin extracts based on four key phenolics (Euclidean distance, Ward's method). Clustering was based on the concentrations of gallic acid, pyrogallol, catechin, and taxifolin—identified as top contributors to antioxidant activity. UAE–EtOH formed a distinct cluster, highlighting its unique phenolic signature.

Table S11. Antibacterial activity of *P. lentiscus* L. resin extracts obtained by CM, SE, and UAE, sequentially using acetone and 70% EtOH as solvents. Inhibition zone diameters (mm) and minimum inhibitory/bactericidal concentrations (MIC/MBC, %) against selected bacterial strains.

**Extraction type**	**Solvent**	***S. aureus***	***M. luteus***	***E. coli***	***P. aeruginosa***
		**Inhibition zone (mm)**			
CM	Acetone	11.0 ± 0.2	10.0 ± 0.1	9.0 ± 0.1	10.0 ± 0.2
	70% EtOH	11.5 ± 0.1	12.3 ± 0.1	11.0 ± 0.3	12.4 ± 0.4
SE	Acetone	10.5 ± 0.1	11.5 ± 0.1	13.0 ± 0.1	9.0 ± 0.3
	70% EtOH	10.0 ± 0.1	13.0 ± 0.2	15.0 ± 0.2	12.0 ± 0.1
UAE	Acetone	10.0 ± 0.4	11.0 ± 0.2	10.0 ± 02	11.0 ± 0.2
	70% EtOH	10.0 ± 0.4	10.0 ± 0.2	10.0 ± 0.4	10.0 ± 0.2
Gentamicin		19.5 ± 0.2	21.5 ± 0.4	22.5 ± 0.4	20.5 ± 0.3
		**MIC/MBC (%)**			
CM	Acetone	n.d. / n.d.	n.d. / n.d.	n.d. / n.d.	n.d. / n.d.
	70% EtOH	n.d. / n.d.	n.d. / n.d.	n.d. / n.d.	n.d. / n.d.
SE	Acetone	n.d. / n.d.	n.d. / n.d.	n.d. / n.d.	n.d. / n.d.
	70% EtOH	n.d. / n.d.	n.d. / n.d.	n.d. / n.d.	n.d. / n.d.
UAE	Acetone	n.d. / n.d.	n.d. / n.d.	n.d. / n.d.	n.d. / n.d.
	70% EtOH	n.d. / n.d.	n.d. / n.d.	n.d. / n.d.	n.d. / n.d.

Note: Inhibition zones are expressed as mean ± standard deviation (n = 3). MIC and MBC values are reported as "n.d." (not detected) for all extracts within the tested concentration range (1.7%–13.3%). Gentamicin was used as the reference antibiotic.

Table S12. Antifungal activity of *P. lentiscus* L. resin extracts obtained by CM, SE, and UAE, each performed sequentially using acetone and 70% EtOH as solvents. Inhibition zone diameters (mm) and minimum inhibitory/fungicidal concentrations (MIC/MFC, %) against selected fungal strains.

Extraction type	Solvent	*G. candidum*	*A. niger*	*C. glabrata*	*C. albicans*	*R. glutinis*
		Inhibition zone (mm)				
CM	Acetone	15.5 ± 0.3	13.0 ± 0.2	15.0 ± 0.1	13.0 ± 0.2	19.5 ± 0.1
	70% EtOH	11.5 ± 0.1	12.3 ± 0.1	11.0 ± 0.3	12.4 ± 0.4	12.4 ± 0.4
SE	Acetone	20.0 ± 0.1	13.0 ± 0.3	14.0 ± 0.1	10.0 ± 0.3	19.0 ± 0.1
	70% EtOH	16.9 ± 0.1	20.0 ± 0.1	16.0 ± 0.3	13.0 ± 0.2	19.5 ± 0.1
UAE	Acetone	11.0 ± 0.4	12.0 ± 0.4	13.0 ± 0.2	14.0 ± 0.3	19.0 ± 0.1
	70% EtOH	12.0 ± 0.2	17.5 ± 0.2	15.5 ± 0.3	13.0 ± 0.2	20.0 ± 0.1
Cycloheximide		19.5 ± 0.2	21.5 ± 0.3	22.5 ± 0.4	20.5 ± 0.2	20.5 ± 0.3
		MIC/MFC (%)				
CM	Acetone	13.3 / n.d.	n.d. / n.d.	13.3 / n.d.	n.d. / n.d.	3.3 / 13.3
	70% EtOH	6.7 / n.d.	6.7 / 13.3	6.7 / n.d.	n.d. / n.d.	3.3 / 13.3
SE	Acetone	1.7/ 13.3	n.d. / n.d.	13.3 / n.d.	n.d. / n.d.	3.3 / 13.3
	70% EtOH	6.7 / n.d.	1.7 / 13.3	6.7 / n.d.	n.d. / n.d.	3.3 / 13.3
UAE	Acetone	n.d. / n.d.	n.d. / n.d.	n.d. / n.d.	13.3 / n.d.	3.3 / 13.3
	70% EtOH	n.d. / n.d.	6.7 / n.d	13.3 / n.d.	n.d. / n.d.	1.7 / 13.3
Cycloheximide		<1.7 / <1.7				

Note: "n.d." indicates no detectable activity at the tested concentrations. Cycloheximide was used as the reference antibiotic.

Table S13. Two-way ANOVA results for the antifungal activity of *P. lentiscus* L. resin extracts against *G. candidum*, *A. niger*, and *R. glutinis strains*. The analysis evaluates the effects of extraction method and solvent on inhibition zones (mm) for each strain.

**Strain**	**Source**	**sum_sq**	**df**	**F**	**PR(>F)**
G. candidum	C(Extraction)	149.7578	2	921.1684	7.34E-14
	C(Solvent)	18.4131	1	226.52	3.74E-09
	C(Extraction):C(Solvent)	23.46379	2	144.327	4.04E-09
	Residual	0.975442	12		
A. niger	C(Extraction)	44.78384	2	553.5985	1.52E-12
	C(Solvent)	70.62666	1	1746.113	2.29E-14
	C(Extraction):C(Solvent)	51.60156	2	637.8763	6.55E-13
	Residual	0.485375	12		
R. glutinis	C(Extraction)	46.57496	2	1102.65	2.51E-14
	C(Solvent)	15.46341	1	732.1845	3.99E-12
	C(Extraction):C(Solvent)	60.56818	2	1433.936	5.23E-15
	Residual	0.253435	12		

Table S14. Tukey HSD pairwise comparisons of inhibition zones (mm) for *G. candidum*, *A. niger*, and *R. glutinis* treated with *P. lentiscus* L. resin extracts.

Strain	Comparison	Mean Diff. (mm)	p-value	Significant
G. candidum	CM-Acetone vs CM-EtOH	-4.13	< 0.001	Yes
G. candidum	CM-Acetone vs SE-Acetone	4.43	< 0.001	Yes
G. candidum	CM-Acetone vs SE-EtOH	1.33	0.001	Yes
G. candidum	CM-Acetone vs UAE-Acetone	-4.54	< 0.001	Yes
G. candidum	CM-Acetone vs UAE-EtOH	-3.39	< 0.001	Yes
G. candidum	CM-EtOH vs SE-Acetone	8.55	< 0.001	Yes
G. candidum	CM-EtOH vs SE-EtOH	5.46	< 0.001	Yes
G. candidum	CM-EtOH vs UAE-Acetone	-0.41	0.517	No
G. candidum	CM-EtOH vs UAE-EtOH	0.74	0.068	No
G. candidum	SE-Acetone vs SE-EtOH	-3.09	< 0.001	Yes
G. candidum	SE-Acetone vs UAE-Acetone	-8.97	< 0.001	Yes
G. candidum	SE-Acetone vs UAE-EtOH	-7.81	< 0.001	Yes
G. candidum	SE-EtOH vs UAE-Acetone	-5.87	< 0.001	Yes
G. candidum	SE-EtOH vs UAE-EtOH	-4.72	< 0.001	Yes
G. candidum	UAE-Acetone vs UAE-EtOH	1.15	0.004	Yes
A. niger	CM-Acetone vs CM-EtOH	-0.8	0.004	Yes
A. niger	CM-Acetone vs SE-Acetone	0.06	0.999	No
A. niger	CM-Acetone vs SE-EtOH	6.87	< 0.001	Yes
A. niger	CM-Acetone vs UAE-Acetone	-1.33	< 0.001	Yes
A. niger	CM-Acetone vs UAE-EtOH	4.54	< 0.001	Yes
A. niger	CM-EtOH vs SE-Acetone	0.85	0.002	Yes
A. niger	CM-EtOH vs SE-EtOH	7.67	< 0.001	Yes
A. niger	CM-EtOH vs UAE-Acetone	-0.53	0.06	No
A. niger	CM-EtOH vs UAE-EtOH	5.33	< 0.001	Yes
A. niger	SE-Acetone vs SE-EtOH	6.81	< 0.001	Yes
A. niger	SE-Acetone vs UAE-Acetone	-1.39	< 0.001	Yes
A. niger	SE-Acetone vs UAE-EtOH	4.48	< 0.001	Yes
A. niger	SE-EtOH vs UAE-Acetone	-8.2	< 0.001	Yes
A. niger	SE-EtOH vs UAE-EtOH	-2.33	< 0.001	Yes
A. niger	UAE-Acetone vs UAE-EtOH	5.87	< 0.001	Yes
R. glutinis	CM-Acetone vs CM-EtOH	-7.03	< 0.001	Yes
R. glutinis	CM-Acetone vs SE-Acetone	-0.41	0.04	Yes
R. glutinis	CM-Acetone vs SE-EtOH	0.01	1	No
R. glutinis	CM-Acetone vs UAE-Acetone	-0.53	0.007	Yes
R. glutinis	CM-Acetone vs UAE-EtOH	0.51	0.01	Yes
R. glutinis	CM-EtOH vs SE-Acetone	6.62	< 0.001	Yes
R. glutinis	CM-EtOH vs SE-EtOH	7.04	< 0.001	Yes
R. glutinis	CM-EtOH vs UAE-Acetone	6.5	< 0.001	Yes
R. glutinis	CM-EtOH vs UAE-EtOH	7.54	< 0.001	Yes
R. glutinis	SE-Acetone vs SE-EtOH	0.43	0.034	Yes
R. glutinis	SE-Acetone vs UAE-Acetone	-0.12	0.91	No
R. glutinis	SE-Acetone vs UAE-EtOH	0.92	< 0.001	Yes
R. glutinis	SE-EtOH vs UAE-Acetone	-0.54	0.006	Yes
R. glutinis	SE-EtOH vs UAE-EtOH	0.5	0.012	Yes
R. glutinis	UAE-Acetone vs UAE-EtOH	1.04	< 0.001	Yes

Table S15. Spearman correlation coefficients (ρ) between selected phenolic compounds of *P. lentiscus* L. resin extracts and antifungal activity (inhibition zone, mm) against three fungal strains.

Compound	*A. niger*	*G. candidum*	*R. glutinis*
Apigenin	-0.515	0.029	-0.403
Caffeic acid	-0.058	-0.6	0.147
Catechin	-0.058	-0.714	0.147
Chlorogenic acid	0.203	0.771	-0.147
Chrysin	0.29	-0.143	0.853*
Curcumin	-0.544	-0.754	-0.537
Ellagic acid	-0.058	0.257	-0.412
Epicatechin	-0.232	0.2	-0.383
Epigallocatechin gallate	-0.626	-0.772	-0.302
Ferulic acid	-0.456	-0.406	-0.224
Galangin	-0.721	-0.696	-0.612
Gallic acid	0.203	-0.257	0.412
Hesperidin	0.721	0.029	0.612
Isorhamnetin	-0.754	-0.143	-0.588
Luteolin	0.377	-0.086	0.383
Myricetin	-0.5	-0.899*	-0.254
Naringenin	0.145	-0.086	0.736
Naringin	0.294	0.261	-0.343
Peonidin-3-*O*-glucoside	-0.059	0.232	0.194
Pyrogallol	0.406	-0.257	0.441
Quercetin	-0.203	0.257	-0.412
Resveratrol	-0.493	-0.143	-0.883*
Rosmarinic Acid	-0.232	-0.429	0.412
Sinapic acid	0.203	0.486	-0.265
Syringic acid	-0.029	-0.371	-0.118
Taxifolin	0.203	-0.257	0.412
Vanillic acid	-0.232	-0.771	-0.147
Vitexin	0.647	0.986*	0.313
p-Coumaric acid	-0.088	-0.754	0.194
p-Hydroxybenzoic acid	0.203	-0.371	0.088

Note: Significant correlations (∣ρ∣ ≥ 0.6, $p < 0.05$) are marked with an asterisk.

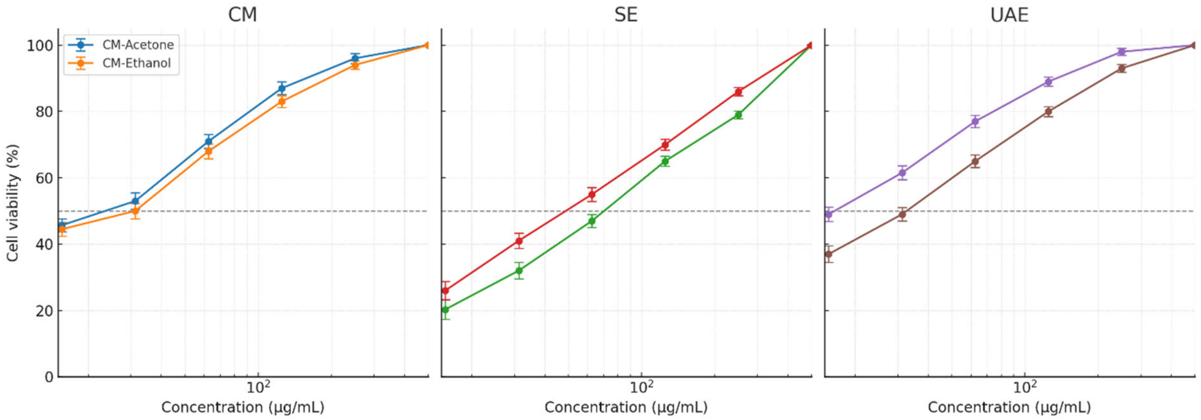

Figure S5. Dose–response cytotoxicity curves of *Pistacia lentiscus* L. resin extracts obtained by CM, SE, and UAE, using acetone and 70% EtOH as solvents. Cell viability was assessed after 24 h treatment of MIA PaCa-2 cells via WST-8 assay. Data are expressed as mean ± SD (n = 3). Concentration range: 50–500 µg/mL.

Table S16. Two-way ANOVA results for the cytotoxic activity of *P. lentiscus* L. resin extracts against MIA PaCa-2 human pancreatic cancer cell. The analysis evaluates the effects of extraction method and solvent on inhibition on IC_{50} (μg/mL) for each strain.

**Source**	**sum_sq**	**df**	**F**	**PR(>F)**
C(Method)	5499.712	2	210.2296	4.56E-10
C(Solvent)	8671.128	1	662.9176	7.18E-12
C(Method):C(Solvent)	2490.047	2	95.18346	4.35E-08
Residual	156.963	12		

Table S17. Tukey HSD pairwise comparisons of cytotoxicity (IC_{50}, mg/mL) of *P. lentiscus* L. resin extracts against MIA PaCa-2 human pancreatic cancer cell.

**Comparison**	**Mean Diff. (μg/mL)**	***p*-value**	**Significant**
CM–acetone vs CM–ethanol	76.09	<0.001	Yes
CM–acetone vs SE–acetone	-13.91	<0.001	Yes
CM–acetone vs SE–ethanol	6.95	<0.001	Yes
CM–acetone vs UAE–acetone	8.14	<0.001	Yes
CM–acetone vs UAE–ethanol	43.2	<0.001	Yes
CM–ethanol vs SE–acetone	-90	<0.001	Yes
CM–ethanol vs SE–ethanol	-69.14	<0.001	Yes
CM–ethanol vs UAE–acetone	-67.95	<0.001	Yes
CM–ethanol vs UAE–ethanol	-32.89	<0.001	Yes
SE–acetone vs SE–ethanol	20.86	<0.001	Yes
SE–acetone vs UAE–acetone	22.05	<0.001	Yes
SE–acetone vs UAE–ethanol	57.11	<0.001	Yes
SE–ethanol vs UAE–acetone	1.19	0.998	No
SE–ethanol vs UAE–ethanol	36.25	<0.001	Yes
UAE–acetone vs UAE–ethanol	35.06	<0.001	Yes
